# Therapeutic targeting with DABIL‐4 depletes myeloid suppressor cells in 4T1 triple‐negative breast cancer model

**DOI:** 10.1002/1878-0261.12938

**Published:** 2021-03-24

**Authors:** Sadiya Parveen, Sumit Siddharth, Laurene S. Cheung, Alok Kumar, Jessica Shen, John R. Murphy, Dipali Sharma, William R. Bishai

**Affiliations:** ^1^ Department of Medicine Division of Infectious Diseases Johns Hopkins University School of Medicine Baltimore MD USA; ^2^ Department of Oncology Sidney Kimmel Comprehensive Cancer Center at Johns Hopkins Johns Hopkins University School of Medicine Baltimore MD USA

**Keywords:** DABIL‐4, diphtheria fusion toxin, IL‐4R, myeloid‐derived suppressor cells, triple‐negative breast cancer, tumor‐associated macrophages

## Abstract

In many solid tumors including triple‐negative breast cancer (TNBC), upregulation of the interleukin‐4 receptor (IL‐4R) has been shown to promote cancer cell proliferation, apoptotic resistance, metastatic potential, and a Th2 response in the tumor microenvironment (TME). Since immunosuppressive cells in the TME and spleen including myeloid‐derived suppressor cells (MDSCs) and tumor‐associated macrophages (TAMs) also express the IL‐4R, we hypothesized that selective depletion of IL‐4R‐bearing cells in TNBC would result in the direct killing of tumor cells and the depletion of immunosuppressive cells and lead to an enhanced antitumor response. To selectively target IL‐4R^+^ cells, we employed DABIL‐4, a fusion protein toxin consisting of the catalytic and translocation domains of diphtheria toxin fused to murine IL‐4. As anticipated, DABIL‐4 has potent cytotoxic activity against TNBC cells both *in vitro* and *in vivo*. We demonstrate in the murine 4T1 TNBC model that DABIL‐4 significantly reduces tumor growth, splenomegaly, and lung metastases. Importantly, we also show that the administration of DABIL‐4 results in the selective depletion of MDSCs, TAMs, and regulatory T cells in treated mice, with a concomitant increase in IFN‐γ^+^ CD8 effector T cells in the TME. Since the 4T1 antitumor activity of DABIL‐4 was largely diminished in IL‐4R knockout mice, we postulate that DABIL‐4 functions primarily as an immunotherapeutic by the depletion of MDSCs, TAMs, and regulatory T cells. NanoString analysis of control and treated tumors confirmed and extended these observations by showing a marked decline of mRNA transcripts that are associated with tumorigenesis and metastasis. In conclusion, we demonstrate that DABIL‐4 targeting of both tumor and immunosuppressive host cells likely represents a novel and effective treatment strategy for 4T1 TNBC and warrants further study.

## Graphical abstract

Treatment with the interleukin‐4 diphtheria fusion toxin protein (DABIL‐4) targeted and killed both tumor cells and the immunosuppressive cell populations of the tumor microenvironment. DABIL‐4 treatment in a murine triple‐negative breast cancer (TNBC) model reduced MDSCs, tumor‐associated macrophages, and Treg cell populations and inhibited both tumor growth and metastasis. DABIL‐4 targeting likely represents a novel and effective treatment strategy for TNBC.

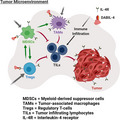

**Abbreviations**
Bregs: regulatory B cells
DT: diphtheria toxin
GzmK: Granzyme K
IC_50_: half‐maximal inhibitory concentration
IFN‐γ: interferon‐gamma
IL‐10: interleukin‐10
IL‐4: Interleukin‐4
IL‐4R: Interleukin‐4 receptor
KO: knockout
Luc: luciferase
MDSCs: myeloid‐derived suppressor cells
M‐MDSCs: monocytic myeloid‐derived suppressor cells
Mɸ: macrophages
NK cells: natural killer cells
PMN‐MDSCs: polymorphonuclear myeloid‐derived suppressor cells
TAMs: tumor‐associated macrophages
TME: tumor microenvironment
TNBC: triple‐negative breast cancer
TNF‐α: tumor necrosis factor‐alpha
Tregs: regulatory T cells

## 1 Introduction

Interleukin 4 (IL‐4) is an important pleiotropic cytokine primarily secreted by activated Th2 lymphocytes, basophils, mast cells, and eosinophils. IL‐4 promotes differentiation of Th2 cells, upregulates the expression of major histocompatibility complexes and the IL‐4 receptor (IL‐4R), and regulates immunoglobulin class switching, especially immunoglobulin E production, making it an important cytokine in allergic responses [1]. IL‐4 functions by binding to three different classes of IL‐4R. The type I receptor is primarily found on T cells, basophils, mast cells, natural killer cells (NK cells), and most B cells and consists of the IL‐4Rα and common gamma C (γc) subunits. The type II receptor, comprised of the IL‐13Rα1 and IL‐4Rα subunits, is expressed on tumor cells. And the type III receptor is present on B cells and monocytes, consists of the IL‐4Rα, IL‐13Rα1, and γc subunits [2].

Upregulation of the IL‐4R (CD124) has been shown in multiple human and murine malignancies including glioma, lung, breast, pancreatic, bladder, colon, and ovarian cancers [2]. The binding of IL‐4 to the IL‐4R recruits and phosphorylates tyrosine kinases Jak1/2 and Tyk2, which then activate the PI3K/AKT, mitogen‐activated protein kinase (MAPK), and Jak/STAT6 signaling pathways [3]. Upon activation, these pathways promote cancer cell proliferation, resistance to apoptosis, and metastatic potential [3, 4]. In the tumor microenvironment (TME), IL‐4 elicits an increased Th2 response and activates myeloid‐derived suppressor cells (MDSCs) and tumor‐associated macrophages (TAMs), both of which express the IL‐4R, to promote immunosuppression and angiogenesis [2]. Therapeutic targeting of the IL‐4/IL‐4R interaction and its consequences has led to effective antitumor strategies, which include pharmacological inhibition of the downstream pathways (AKT, ERK, JAK/STAT6, and mTOR), monoclonal antibody blockade of IL‐4 or the IL‐4R, engineered IL‐4R antagonists, and IL‐4 fused with cytotoxic payloads [2, 3].

In breast cancer, upregulation of IL‐4/IL‐4R signaling has been associated with poor prognosis in both human and murine models [5]. Additionally, IL‐4 blockade has been shown to effectively downregulate the MAPK pathway and reduce the growth and invasion of breast cancer cells [4, 6]. Interestingly, triple‐negative breast cancer (TNBC) cells were shown to secrete higher levels of IL‐4 in the tumor milieu, compared with estrogen receptor (ER)‐positive breast cancer cells, contributing to their proliferation and metastatic potential [4], and recently, an IL‐4 mediated boost in glucose and glutamine metabolism was identified as a driver of TNBC cell growth [7]. As TNBC tumors are difficult to treat owing to their inherent molecular heterogeneity and lack of targetable receptors (estrogen, progesterone, and HER2) [8, 9, 10], we sought to interrupt the IL‐4/IL‐4R signaling axis in TNBC using a targeted bacterial protein toxin‐based approach.

In the present study, we have determined the underlying mechanistic details of the action of IL‐4R‐targeted fusion protein toxin: s‐DAB_1–389_‐mIL‐4 (referred to as DABIL‐4 hereafter) in a murine model of TNBC adenocarcinoma. DABIL‐4 consists of the catalytic and translocation domains of diphtheria toxin (DT) genetically fused to murine IL‐4 (mIL‐4) [11]. We demonstrate that DABIL‐4 selectively targets the IL‐4R^+^ murine 4T1 TNBC cells *in vitro*. *In vivo* administration of DABIL‐4 in 4T1 tumor‐bearing mice results in significant reductions in tumor growth, splenomegaly, and metastases to lung. We also demonstrate a marked decline in the population of IL‐4R^+^ MDSCs, TAMs, and regulatory T cells (Tregs) in TME and/or spleen that is associated with a concomitant increase in INF‐γ^+^ effector T cells.

## 2 Materials and methods

### 2.1 Plasmids, bacterial strains, and cloning

*Escherichia coli–Corynebacterium diphtheriae* shuttle vector pKN2.6Z LC127 was constructed in the laboratory [12]. Murine IL‐4 sequence was codon‐optimized as per *C. diphtheriae* codon usage table and was synthesized by GenScript. hIL‐2 sequence in pKN2.6z LC127 was replaced with the mIL‐4 sequence generating pKN2.6z LC128 using Gibson assembly (NEB, New England Biolabs, Rowley, MA, USA). Primers were specifically designed to introduce tobacco etch virus protease sequence between murine IL‐4 and His‐6 sequence. The construct was then transformed into either chemically competent *E. coli* DH5α strain (NEB) or electrocompetent *C. diphtheriae* C7s(−)*tox*‐.

### 2.2 Fermenter protein purification

*Corynebacterium diphtheriae* nonlysogenic nontoxigenic C7s(‐)*tox*‐ strain transformed with pKN2.6Z‐LC128 was grown in CY medium in a fermenter (BioFlo/CelliGen 110) as previously described [12]. At OD ~ 12–15, culture was harvested, and the supernatant was collected and concentrated 20‐fold by tangential flow filtration and a 30 kD hollow fiber membrane (Repligen, Waltham, MA, USA). The flow concentrate was then diafiltered against 2 L of 50 mm NaH_2_PO_4_, 500 mm NaCl, and 50 mm imidazole (pH 7.4). The concentrate was then adsorbed onto a HisTrap HP column (Cytiva, Malborough, MA, USA), and the protein was eluted with 50 mm NaH_2_PO_4_, 500 mm NaCl, and 500 mm imidazole (pH 7.4). The eluate was again concentrated using 10 kDa Amicon Ultra‐15 centrifugal units (Millipore Sigma, Burlington, MA, USA) and separated over a HiPrep 26/60 Sephacryl S‐100 HR sizing column (Cytiva). The protein was eluted with PBS in 5 mL fractions and analyzed by SDS/PAGE. The protein concentration was then estimated, and aliquots were stored at −80 °C in PBS.

### 2.3 Cell lines, media, and growth conditions

Murine origin TNBC cell lines 4T1 (laboratory stock) and EO771 (purchased from CH3 Biosystems, Amherst, NY, USA) were grown in DMEM F12 (Corning Life Sciences, Tewksbury, MA, USA) and RPMI‐1640 (Corning) + 0.1% HEPES, respectively, supplemented with 10% heat‐inactivated FBS (Gibco Laboratories, Gaithersburg, MD, USA) and 1% antibacterial antimycotic solution (Sigma‐Aldrich Inc, Miamisburg, OH, USA). The cell lines were grown in a humidified incubator at 37 °C under 5% CO_2_ atmosphere.

### 2.4 MTS cell viability assay

For MTS assays, 5000 4T1 cells were plated per well in triplicate in 200 μL volume in 96‐well plates, allowed to adhere, and treated with twofold serial dilutions of DABIL‐4 fusion toxin or no drug. After 48‐h incubation with the drug, 3‐(4,5‐dimethylthiazol‐2‐yl)‐5‐(3‐carboxymethoxyphenyl)‐2‐(4‐sulfophenyl)‐2H‐tetrazolium, inner salt; MTS reagent (Promega Corporation, Madison, WI, USA) was added to the individual wells. After 90–120 min, absorbance at 490 nm was recorded with the iMark Microplate Reader (Bio‐Rad, Hercules, CA, USA) to score cell viability.

### 2.5 Western blots

For analyzing the protein samples, cell lysate or purified protein was boiled in 4× SDS loading buffer (Bio‐Rad) and separated on precasted 4–15% SDS/PAGE gel (Bio‐Rad). The separated proteins were transferred to activated poly(vinylidene difluoride) membrane and probed with appropriate primary antibodies followed by secondary antibodies and detected by ECL Reagent (Thermo Fisher Scientific, Waltham, MA, USA). Antibodies for western blots were purchased from different sources: anti‐IL‐2 (CST, Danvers, MA, USA, Cat# 12239), anti‐His (Abcam, Cat# ab18184), anti‐DT (Abcam, Cambridge, MA, USA, Cat# ab53828), anti‐total PARP (CST, Cat# 9532), anti‐cleaved PARP (CST, Cat# 5625), anti‐BCL‐xL (CST, Cat#), anti‐cleaved caspase‐3 (CST, Cat#), and anti‐β‐actin (Sigma, Cat# A5441). CST stands for Cell Signaling Technology.

### 2.6 Caspase activity assay

4T1 cells were seeded at the density of 1000 cells per well. After adherence, cells were treated with no drug, 20 nm DABIL‐4, and 2.5 µM doxorubicin (as a positive control). Caspase activities in cells were quantified after 48 h of treatment using Caspase‐Glo® 3/7 Assay System (Promega) as per manufacturer's instructions.

For testing the expression of apoptosis‐associated markers (such as PARP, caspase‐3, and BCL‐xl), 4T1 cells were seeded in six‐well plates at a density of 5000 cells per well and treated with DABIL‐4 or no drug. After 48 h, cells were trypsinized, washed with PBS, and lysed with RIPA buffer (Thermo Fisher) for 30 min on ice. The lysates were then centrifuged at 15 800 g for 10 min at 4 °C. The protein concentration was estimated as per Bradford assay, and equal samples were loaded on SDS/PAGE gel (40 μg protein per lane). Western blot was performed as mentioned in the earlier section.

### 2.7 *In vivo* mouse experiments

All animal experimental procedures and protocols were reviewed and approved by Johns Hopkins University Animal Care and Use Committee. The animals were housed at the animal vivarium located in Koch Cancer Research Building located at the East Baltimore Campus of Johns Hopkins University. For tumor generation, 10 000 4T1 cells in 0.1 mL Matrigel (50% v/v in PBS; Corning) were orthotopically implanted into fourth mammary fat pad of 6‐week‐old female Balb/c mice (Charles River Laboratories, Wilminton, MA, USA) and Balb/c IL‐4R knockout (KO) mice (purchased from Jackson Laboratory, Bar Harbor, ME, USA). On day 7, when tumor became palpable, PBS and DABIL‐4 (10 µg per mice in 100 µL PBS) were administered *via* intraperitoneal route on alternate days thrice a week till the completion of the study. Tumor growth was assessed twice a week (Monday and Thursday) by measuring tumor volume using electronic caliper. Tumor volume was calculated using the following equation = length × width × width × 0.5. Mice were sacrificed at the indicated time points. Tumor and spleen were isolated and weighed.

All animal experimental procedures and protocols were reviewed and approved by Johns Hopkins University Animal Care and Use Committee. The animals were housed at the animal vivarium located in Koch Cancer Research Building located at the East Baltimore Campus of Johns Hopkins University. For tumor generation, 10 000 4T1 cells in 0.1 mL Matrigel (50% v/v in PBS; Corning) were orthotopically implanted into fourth mammary fat pad of 6‐week‐old female Balb/c mice (Charles River Laboratories, Wilminton, MA, USA) and Balb/c IL‐4R knockout (KO) mice (purchased from Jackson Laboratory, Bar Harbor, ME, USA). On day 7, when tumor became palpable, PBS and DABIL‐4 (10 µg per mice in 100 µL PBS) were administered *via* intraperitoneal route on alternate days thrice a week till the completion of the study. Tumor growth was assessed twice a week (Monday and Thursday) by measuring tumor volume using electronic caliper. Tumor volume was calculated using the following equation = length × width × width × 0.5. Mice were sacrificed at the indicated time points. Tumor and spleen were isolated and weighed.

All animal experimental procedures and protocols were reviewed and approved by Johns Hopkins University Animal Care and Use Committee. The animals were housed at the animal vivarium located in Koch Cancer Research Building located at the East Baltimore Campus of Johns Hopkins University. For tumor generation, 10 000 4T1 cells in 0.1 mL Matrigel (50% v/v in PBS; Corning) were orthotopically implanted into fourth mammary fat pad of 6‐week‐old female Balb/c mice (Charles River Laboratories, Wilminton, MA, USA) and Balb/c IL‐4R knockout (KO) mice (purchased from Jackson Laboratory, Bar Harbor, ME, USA). On day 7, when tumor became palpable, PBS and DABIL‐4 (10 µg per mice in 100 µL PBS) were administered *via* intraperitoneal route on alternate days thrice a week till the completion of the study. Tumor growth was assessed twice a week (Monday and Thursday) by measuring tumor volume using electronic caliper. Tumor volume was calculated using the following equation = length × width × width × 0.5. Mice were sacrificed at the indicated time points. Tumor and spleen were isolated and weighed.

For single‐cell suspension preparation, tumors were minced and digested with collagenase D and DNase I at 37 °C for 1 h, while spleens were passed through 100‐µm mesh filters. Red blood cells were lysed using RBC lysis buffer (BioLegend, San Diego, CA, USA).

### 2.8 Flow cytometry

Single‐cell suspensions from the tumor and spleen were stained with trypan blue and manually scored for viability. One million cells were incubated with purified anti‐mouse CD16/32 antibody (BioLegend, Cat# 101320) in FACS buffer (eBiosciences, San Diego, CA, USA, Cat# 422226) and stained with two panels of antibodies (BioLegend unless otherwise indicated): (a) lymphoid panel: APC/Cy7 CD45, BV785 CD3, BUV563 CD4 (Becton Dickinson, Franklin Lakes, NJ, USA), AF700 CD8a, BV711 CD25, PE‐Texas Red CD39, BV650 CD44, and BV421 PD‐L1; and (b) myeloid panel: APC/Cy7 CD45, BV650 CD11b, BV605 Ly6G (BD), PE/CY7 Ly6C, BV711 CD115, PE CD124, BUV396 IA/IE (BD), APC F4/80, and FITC CD86. For identifying IL‐4R^+^ T, B, and NK cells, a single panel consisting of APC/Cy7 CD45, BV785 CD3, BUV563 CD4 (BD), AF700 CD8a, BV650 CD11b, APC CD19, PE/Cy7 CD49b, BV605 Ly6G, and PE CD124 was used. Zombie Aqua fixable viability dye was included in both the panels to select viable cells. After surface staining, cells were fixed in fixation buffer and intracellular staining was performed using transcription factor buffer set followed by staining with FITC Foxp3 (lymphoid panel) and PerCP/Cy5.5 CD206 (myeloid panel). The stained samples were acquired on LSRFortessa™ X‐20 Cell Analyzer (BD), and data were analyzed using flowjo v.10 software (BD). To exclude debris and aggregates, FSC‐A versus SSC‐A gate, and to exclude duplets, FSC‐W and SSC‐H gates were designed. Inside this latter gate, dead cells were excluded using the Zombie Red fixable viability dye. All reagents were purchased from BioLegend unless otherwise indicated.

### 2.9 T‐cell stimulation and intracellular cytokine staining

For *in vitro* stimulations, harvested cells were incubated with cell activation cocktail (PMA/ionomycin without Brefeldin) and monensin in RPMI‐1640 media supplemented with 10% FBS at 37 C for 4 h. After stimulation, cells were surface‐stained with APC/Cy7 CD45, BV785 CD3, BUV563 CD4, and AF700 CD8a, as described earlier. Intracellular cytokine staining was performed with Cyto‐Fast™ Fix/Perm Buffer Set (BioLegend) as per the manufacturer's protocol and labeled with BV421 interferon‐gamma (IFN‐γ) or PE IL‐10. Samples were acquired on LSRFortessa™ X‐20 Cell Analyzer (BD), and data were analyzed using flowjo v.10 software (BD). Zombie Aqua fixable dye was included to score viability. All reagents were supplied by BioLegend unless otherwise indicated.

### 2.10 Lung metastasis assay

To quantify metastases, lungs were isolated from tumor‐bearing mice, excised, minced, and digested with collagenase D and DNase I to prepare a single‐cell suspension. All the cells from one single lung were plated in one well of six‐well plates in 2 mL volume of DMEM F‐12 media supplemented with *S*‐guanidine and incubated in a humidified incubator at 37 under 5% CO_2_ for 10–14 days. Following selection, colonies were formalin‐fixed and stained with crystal violet prior to manual counting. Plates were also photographed for record keeping.

### 2.11 IVIS imaging‐based metastasis assay

10 000 4T1‐Luciferase (Luc) cells were orthotopically implanted into fourth mammary fat pads of syngeneic Balb/c mice (*n* = 3; purchased from Charles River Laboratories) [13]. On day 12, primary tumors were removed and mice were distributed into two groups. PBS and 10 µg DABIL‐4 were administered i.p. to groups 1 and 2, respectively. On day 9, the mice were injected with 0.15 g·Kg^−1^ d‐luciferin (Sigma‐Aldrich) and were imaged within 10 min using IVIS Imaging System (Perkin Elmer, Hopkinton, MA, USA). The images were analyzed using IVIS imaging software by Perkin Elmer. During image analysis, total photon flux coming from thoracic cavity was quantified and plotted.

### 2.12 Immunohistochemistry assay

At the specified time point, 4T1 tumors were harvested and fixed in 10% formalin. The fixed sections were then paraffin‐embedded and sectioned, and immunohistochemical analyses were performed using anti‐cleaved caspase‐3 (CST, Cat# 9661) antibody. Images were captured using a Leica microscope at 20× magnification.

### 2.13 RNA isolation and NanoString analysis

RNA was isolated from 3 4T1 tumors from both PBS‐treated and DABIL‐4‐treated group by using RNeasy Mini Kit (Qiagen, Germantown, MD, USA) as per manufacturer's protocol. All RNA samples passed quality control (assessed by OD 260/280) and were analyzed by nCounter murine PanCancer Immune Profiling Panel according to manufacturer's protocol at Johns Hopkins Transcriptomics and Deep Sequencing Core Facility (NanoString Technologies, Seattle, WA, USA). Raw data were normalized using the nsolver 3.0 analysis software (NanoString Technologies). Gene expression (represented in log_2_) was calculated as the mean, and data were imported to graphpad prism software for statistical analysis and graphical representation.

## 3 Results

### 3.1 DABIL‐4 selectively targets IL‐4 receptor‐expressing 4T1 cells *in vitro*

Lakkis *et al*. [11] first described the genetic construction and expression of DABIL‐4 in recombinant *E. coli* more than 30 years ago. In the present study, we used a strategy recently developed in our laboratory to reconstruct the structural gene encoding DABIL‐4 with the native diphtheria *tox* signal sequence, the *tox* promoter, and a mutant *tox* operator in recombinant *C. diphtheriae* C7(‐)^tox‐^[12] (Fig. S1). This construct directs the constitutive expression and secretion of DABIL‐4 into culture medium as a fully folded, biologically active, monomeric recombinant protein that is readily purified to > 98% homogeneity (Fig. S2A,B). Immunoblot analysis of the purified protein probed with anti‐DT, anti‐mIL‐4, and anti‐His_6_ antibodies demonstrated the chimeric nature of the fusion toxin (Fig. S2C).

**Fig. 1:**
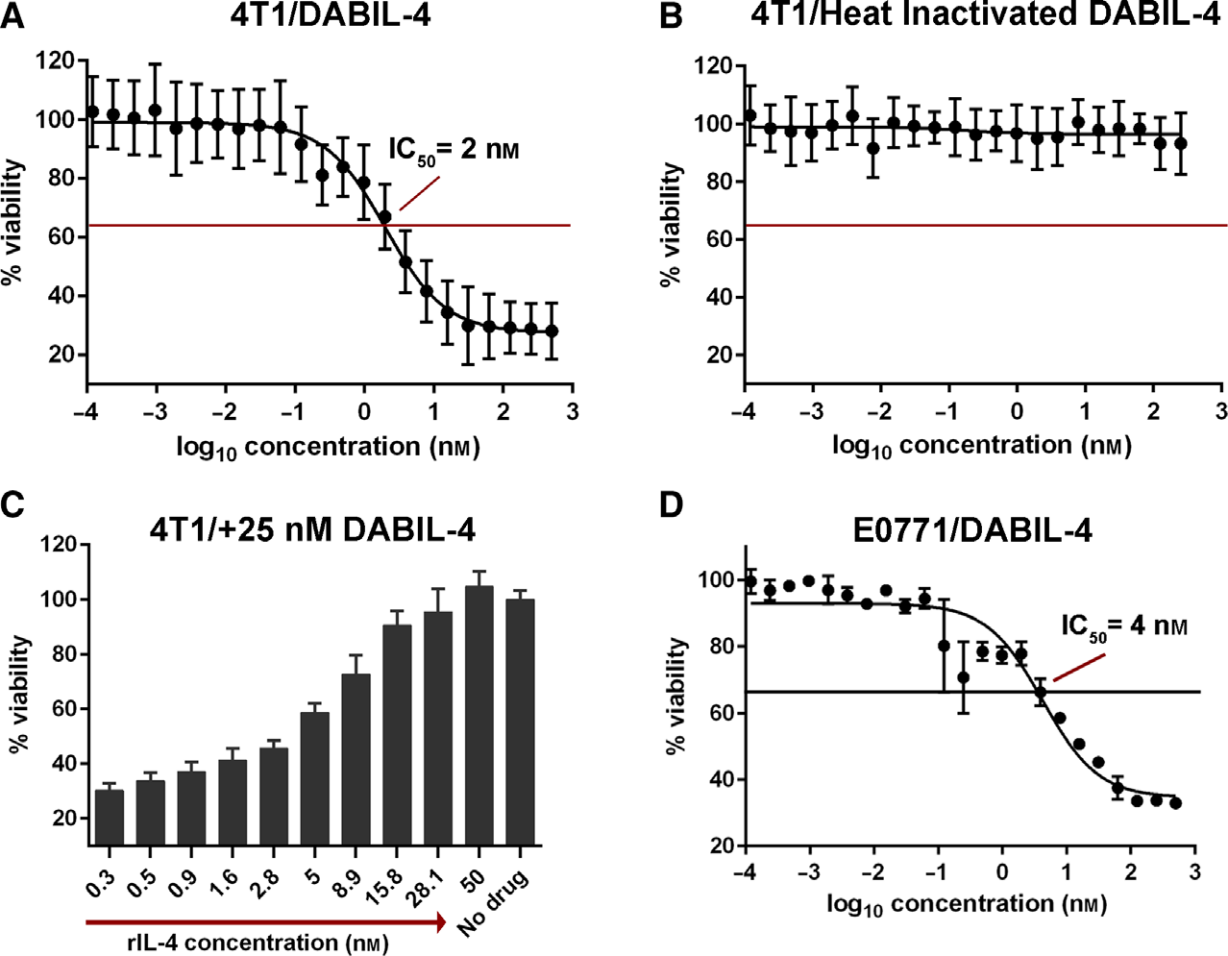
DABIL‐4 exhibits cytotoxic activity against IL‐4R^+^ TNBC cells. (A) Activity of DABIL‐4 against 4T1 cells in an MTS assay showed an IC_50_ of 2 nm, while (B) after heat inactivation, it showed no cytotoxicity. (C) Recombinant IL‐4, in a dose‐dependent fashion, blocked the cytotoxic activity of 25 nm DABIL‐4 in an MTS assay with 4T1. (D) Activity of DABIL‐4 against E0771 cells in an MTS assay showed an IC_50_ of 4 nm. Data are shown as mean ± SD. The experiments were performed in at least triplicates.

To evaluate whether DABIL‐4 was cytotoxic for IL‐4R^+^ TNBC cells, we performed a dose–response analysis using 4T1 cells, a murine IL‐4R^+^ TNBC cell line [6]. Upon incubation with varying concentrations of DABIL‐4 for 48 h, 4T1 cells exhibited sensitivity to DABIL‐4 in a dose‐dependent manner with an half‐maximal inhibitory concentration (IC_50_) of 2 nm (Fig. 1A), while heat‐inactivated DABIL‐4 was nontoxic (Fig. 1B). To demonstrate that the activity of DABIL‐4 is mediated through the IL‐4R, we examined the competitive inhibition of increasing concentrations of recombinant murine IL‐4 upon the cytotoxicity of 25 nm DABIL‐4. As shown in Fig. 1C, recombinant IL‐4 completely blocked the cytotoxic activity of DABIL‐4, demonstrating that the action of the fusion protein is mediated *via* the IL‐4R/IL‐4 interaction. Another TNBC cancer cell line, E0771 [14], also showed sensitivity to the fusion toxin albeit with a slightly higher IC_50_ value of 4 nm, a difference potentially attributable to different IL‐4R expression levels and/or growth rates (Fig. 1D). It should be noted, however, that the TNBC status of E0771 remains controversial [15]. DABIL‐4 also showed cytotoxic activity (IC_50_ ~ 2.3 nm) against NT2.5, a murine transgenic breast cancer cell line, which express rat Her2/neu [16] (Fig. S3A), supporting the potential versatility of this fusion protein toxin.

As previously reported, once internalized by the target cell, the catalytic subunit from either native DT or diphtheria‐based fusion protein toxins is translocated into the cytosol where it catalyzes the ADP‐ribosylation of elongation factor 2 (EF‐2), which leads to the inhibition of cellular protein synthesis [17, 18]. Yamaizumi *et al*. [19] demonstrated that the introduction of a single molecule of the catalytic domain is sufficient to kill a eukaryotic cell, and this occurs within 48 to 72 after exposure by the induction of apoptosis. Using a chemiluminescence‐based assay, we demonstrated that DABIL‐4 treatment led to threefold induction of caspase‐3/7 activity in 4T1 cells, which was comparable to that elicited by the cytotoxic agent doxorubicin (Dox; Fig. S4A). Immunoblot analysis further demonstrated apoptosis induction in DABIL‐4‐treated cells revealing cleavage of both PARP and caspase‐3 (Fig. S4B). These results confirm that DABIL‐4 is a potent cytotoxic agent, which induces apoptosis in cancer cells expressing the IL‐4R.

### 3.2 DABIL‐4 inhibits 4T1 tumor growth and prevents lung metastases *in vivo*

**Fig. 2:**
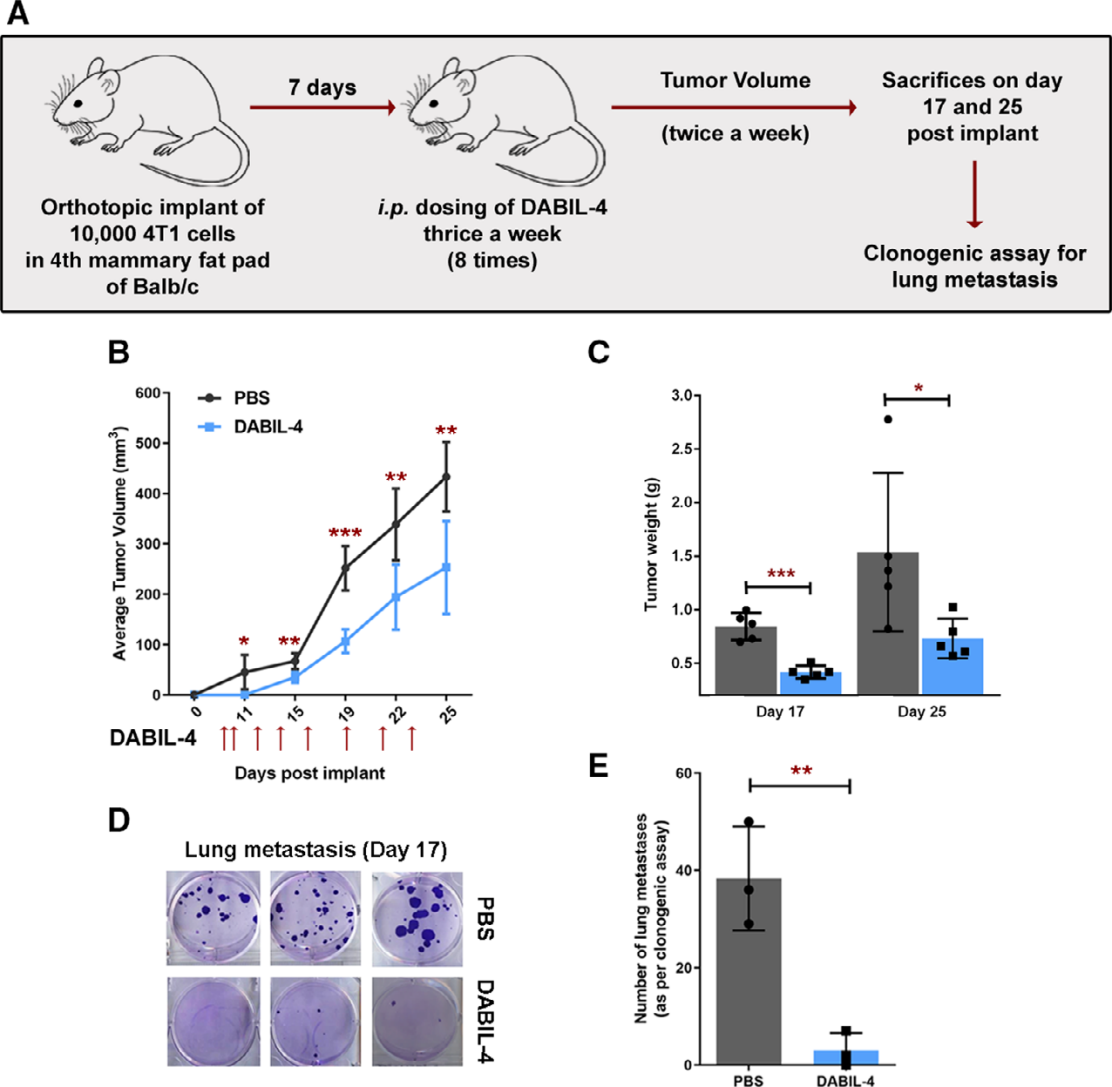
DABIL‐4 shows antitumor activity in 4T1 adenocarcinoma model in Balb/c mice. (A) Schematic of the experiment. Treatment was given thrice weekly on days 7, 9, 12, 14, 16, 19, 21, and 23 post‐tumor implantations. (B) DABIL‐4 administration led to the decline in tumor volume (*n* = 5), (C) tumor weight (*n* = 5), and (D) lung metastasis (*n* = 3) both qualitatively and (E) quantitatively. Statistical significance for tumor growth and lung metastasis experiments was assessed by two‐tailed unpaired Student's *t*‐test considering an unequal distribution. Tumors were measured by electronic Vernier calipers. Red arrows indicate the days when treatment was administered. Data are shown as mean ± SD. *P < 0.05, **P < 0.01, ***P < 0.001.

The 4T1 mammary tumor model in syngeneic BALB/c mice shares many characteristic features with human breast cancer including progressive growth of primary tumors and the ability to metastasize to lungs, liver, bone, and brain. Following orthotopic injection of as few as 10 000 cells into the mammary fat pads, palpable tumors appear within 7 days and metastases are observed by 18–28 days [20]. We utilized the orthotopic 4T1 murine model to evaluate the cytotoxicity of DABIL‐4 against 4T1 cells *in vivo* (Fig. 2A). Tumor‐bearing mice were treated by intraperitoneal injection on alternate days with either 10 μg DABIL‐4 or PBS alone. As shown in Fig. 2B,C, we observed significant reductions in tumor volume and weight in mice treated with DABIL‐4. Upon immunohistochemical staining of the tumors, we also observed a higher expression of caspase‐3 in DABIL‐4‐treated mice compared with the PBS‐treated control (Fig. S5A). Additionally, we isolated tumors, prepared single‐cell suspension, and performed flow cytometry to detect IL‐4R^+^ tumor cells (CD45^−^ CD11b^−^ CD3− CD124^+^) and found that DABIL‐4 treatment significantly reduced the frequency of IL‐4R^+^ cells in tumors (Fig. S5B). The data confirm that DABIL‐4 induces apoptotic cell death in 4T1 tumors *in vivo*. To rule out the strain specificity, we tested the efficacy of DABIL‐4 in the orthotopic E0771 TNBC model generated in C57BL/6 strain background and observed that DABIL‐4 significantly reduced E0771 tumor growth (Fig. S6).

4T1 tumors are also known to have high metastatic potential [20]. To evaluate the impact of DABIL‐4 upon metastatic progression, we harvested lungs from 4T1 tumor‐bearing mice from both treated and untreated mice on day 17, isolated metastatic cancer cells, and performed clonogenic assays. We observed a 12‐fold reduction in the number of metastatic tumor colonies cultured from the lungs of DABIL‐4‐treated mice compared with the PBS‐treated group (Fig. 2D,E). In addition, we confirmed the effect of DABIL‐4 upon metastasis using bioluminescence imaging. Briefly, we orthotopically implanted bioluminescent 4T1‐Luc cells into mammary fat pads, surgically removed primary tumors on day 12, and treated one group of mice with DABIL‐4 every third day. On day 9 post‐tumor removal, we monitored luminescence of the thoracic cavity using the IVIS Imaging System. As shown in Fig. S7A,B, we observed decreased bioluminescence in the thoracic cavities of the DABIL‐4‐treated mice. The thoracic cavities of 2 of the 3 mice from the DABIL‐4‐treated group did not show any bioluminescence.

**Fig. 3:**
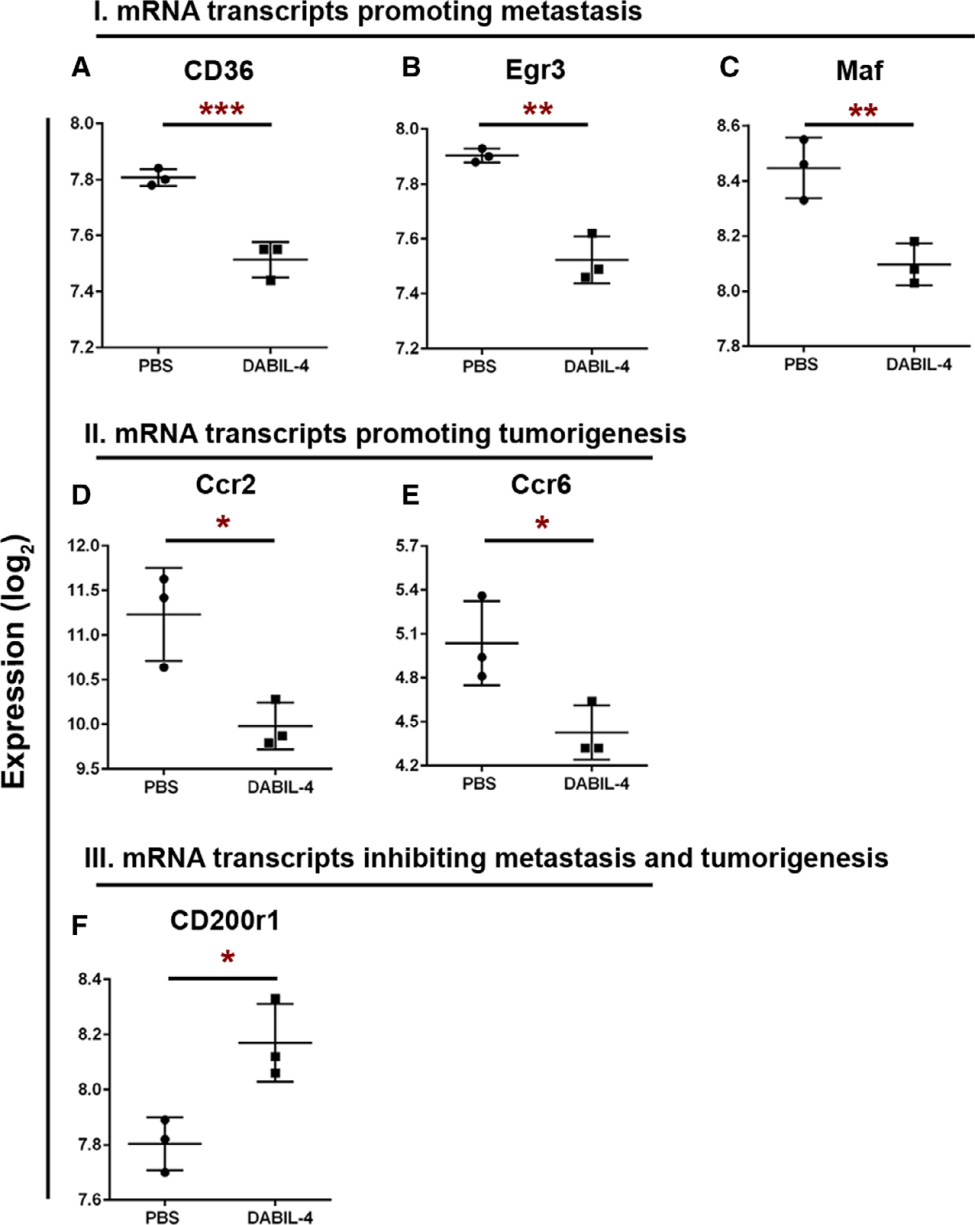
DABIL‐4 monotherapy modulates the level of mRNA transcripts associated with metastasis and/or tumorigenesis in TME. Total RNA was isolated from 4T1 tumors (*n* = 3) from both PBS‐ and DABIL‐4‐treated mice on day 17 and were subjected to NanoString PanCancer Immune Profiling Panel analysis and nSolver software. Expression profiles of transcripts promoting metastasis (A–C) or tumorigenesis (D, E) and transcript inhibiting metastasis and tumorigenesis (F) are shown for both treatment groups. RNA expression values are given as log_2,_ and data are shown as mean ± SD. Statistically significant differences were defined by two‐tailed unpaired Student's *t*‐test considering an unequal distribution. *P < 0.05, **P < 0.01, ***P < 0.001.

To further characterize the *in vivo* effect of DABIL‐4 upon the metastatic potential, we analyzed total RNA isolated from the primary tumors using the NanoString PanCancer Immune Profiling Panel Assay. We noted significant declines in the level of several transcripts associated with metastasis (Cd36 [21], Egr3 [22, 23, 24], and Maf [25]) and tumorigenesis (Ccr2 [26] and Ccr6 [27]) in many solid tumors including breast cancer (Fig. 3 and Table 1). We also observed an increased level of the C200r1 transcript, which is associated with the inhibition of metastasis and tumorigenesis [28, 29] (Fig. 3 and Table 1). These observations indicated that DABIL‐4 not only prevented primary tumor growth, but also inhibited metastatic dissemination of 4T1 cells to secondary sites.

**Table 1:** List of mRNA transcripts identified using NanoString PanCancer immune profiling panel.

mRNA transcript	Expression (log_2_)		*P*‐value	Pathway	Reference
	PBS	s‐DABIL‐4			
Cd36	7.81	7.51	0.005	M	[21]
Egr3	7.90	7.52	0.01	M	[51]
Maf	8.45	8.10	0.01	M	[25]
Ccr2	11.23	9.98	0.04	T	[26]
Ccr6	5.04	4.43	0.04	T	[27]
Cd200r1	7.80	8.17	0.03	T/M	[28, 52]
Gzmk	5.06	6.42	0.04	I	[53]
Il10ra	8.85	8.18	0.04	I	[54]

### 3.3 DABIL‐4 depletes MDSCs and TAMs in the tumor microenvironment and/or spleen

**Fig. 4:**
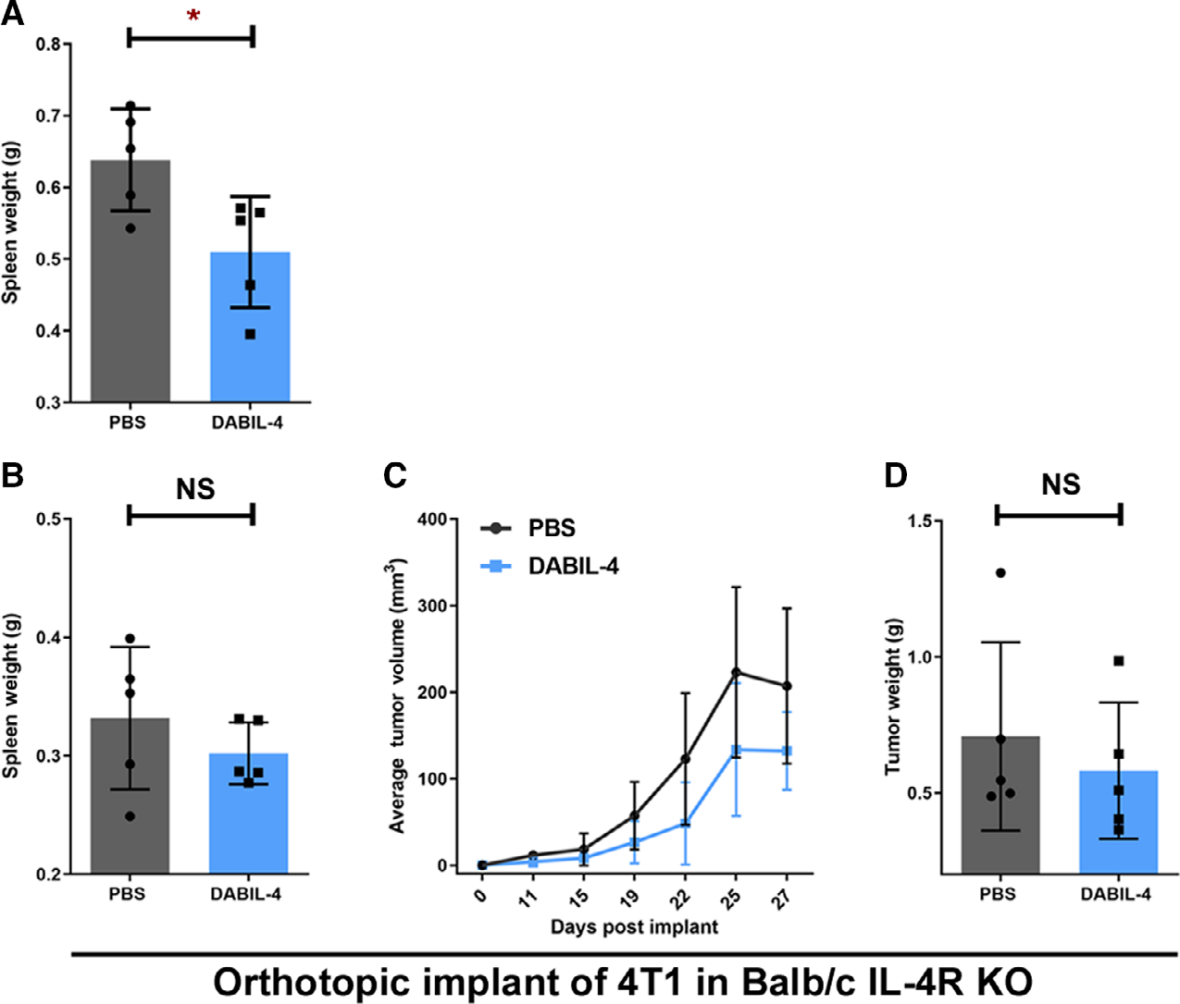
DABIL‐4 shows no antitumor activity against 4T1 adenocarcinoma model in IL‐4R KO mice. (A) DABIL‐4 administration in 4T1 tumor‐bearing Balb/c mice showed reduction in spleen weights. In IL‐4R KO mice, 10 000 4T1 cells were orthotopically implanted and allowed to establish. Starting day 7, DABIL‐4 was given *via* i.p. thrice a week on alternate days for total of 8 doses. Mice were sacrificed on day 27. The fusion toxin administration did not significantly reduce (B) spleen weights, (C) tumor volumes, and (D) tumor weights. Statistical significance for these experiments was assessed by two‐tailed unpaired Student's *t*‐test considering an unequal distribution. Tumors were measured by electronic Vernier calipers. Data are shown as mean ± SD. *P < 0.05, NS = nonsignificant. *N* = 5 mice per group.

In order to further characterize the action of DABIL‐4, we determined its effect on other cells in the TME and spleen. One of the pathologic hallmarks of the 4T1 tumor model is splenomegaly (up to eightfold increases in spleen weight), which is mostly due to granulocytic hyperplasia [30]. We found that DABIL‐4 treatment led to a 20% reduction in the spleen weights in 4T1 tumor‐bearing mice, suggesting that this fusion protein toxin also affects granulocytosis and tumor‐associated leukemoid reactions in addition to tumor growth inhibition (Fig. 4A). It is well known that IL‐4R is not only expressed on 4T1 tumor cells but also expressed on MDSCs and TAMs. Both of these immune cell populations contribute to immunosuppression within the TME and metastasis [31, 32, 33, 34].

To investigate whether the observed inhibition of tumor growth was due to direct targeting of IL‐4R^+^ tumor cells, to depletion of IL‐4R^+^ MDSCs and TAMs, or to a combination of the two, we orthotopically implanted IL‐4R^+^ 4T1 cells in an IL‐4R KO mouse strain (IL‐4Rα^−/−^) and administered either PBS or DABIL‐4 thrice weekly starting on day 7. While we observed a delayed progression of 4T1 tumors in the IL‐4R KO mice, DABIL‐4 treatment was found to reduce spleen weights by only 9% (*P* = 0.35 vs 20% in WT; *P* = 0.02 in WT) and tumor weights by only 18% (*P* = 0.52 vs 52% in WT; *P* = 0.04; Fig. 4B–D). These results strongly suggest that direct killing of 4T1 tumor cells by DABIL‐4 contributes only modestly to the observed delay in tumor expansion. Further, these results suggest that the depletion of IL‐4R^+^ immunosuppressive host cell populations (e.g., MDSCs, TAMs, and Tregs) is most likely the primary mechanism of DABIL‐4's antitumor efficacy in the 4T1 model.

**Fig. 5:**
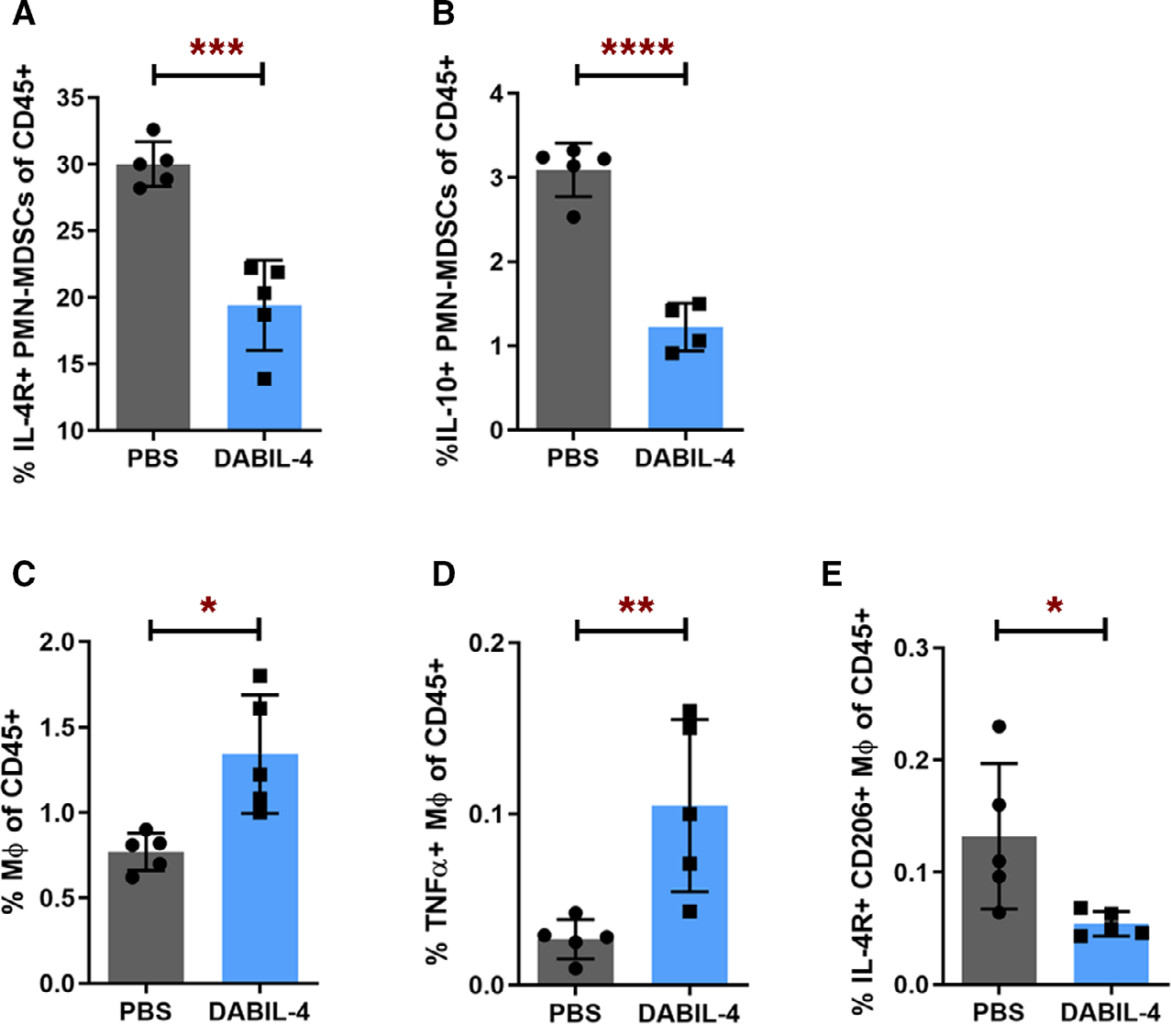
DABIL‐4 administration depletes immunosuppressive myeloid cell populations in spleen. As in Fig. 2A, mice were treated with DABIL‐4 thrice weekly beginning on day 7, and they were sacrificed on day 25. Single‐cell suspensions of spleens from both treatment groups were stained with specific antibodies and analyzed by flow cytometry (*n* = 5). We found differences in the population of (A) IL‐4R^+^ PMN‐MDSCs, (B) IL‐10^+^ PMN‐MDSCs, (C) macrophages, (D) TNF‐α^+^ macrophages, and (E) IL‐4R^+^ CD206^+^ M2 macrophages. Data are represented as mean ± SD and as percentage of CD45^+^ population. Spleen was analyzed on day 25 post‐tumor implant. Statistical significance between the groups was assessed by two‐tailed unpaired Student's *t*‐test considering an unequal distribution. *P < 0.05, **P < 0.01, ***P < 0.001, ****P < 0.0001.

To directly address the effects of DABIL‐4 on host immune cell populations, we analyzed both tumor and spleens from 4T1 tumor‐bearing wild‐type mice using multicolor flow cytometry. As in Fig. 2A, mice began thrice‐weekly DABIL‐4 or PBS treatment on day 7 after 4T1 tumor implantation. We investigated drug's impact on the two major myeloid cell subsets, MDSCs and macrophages. We noted a marked decline in the total MDSC population (Fig. S8A), and evaluation of the two distinct MDSC subsets, polymorphonuclear MDSCs (PMN‐MDSCs; CD11b^+^ Ly6G^+^ Ly6C^low^ CD124^+^) and monocytic MDSCs (M‐MDSCs; CD11b^+^ Ly6G^‐^ Ly6C^high^ CD124^+^), revealed a 60% reduction in IL‐4R^+^ PMN‐MDSC population in DABIL‐4‐treated mice (Fig. 5A and Fig. S9), while the level of IL‐4R^+^ M‐MDSCs remained the same (Fig. S8B). The population of PMN‐MDSC subset expressing IL‐10 also reduced (Fig. 5B). This cytokine is known to skew antitumor T‐cell responses toward a Th2 phenotype and promote tumor growth [35].

We also evaluated the effect of DABIL‐4 on the macrophage population in both the TME and the spleen. As shown in Fig. 5C, we observed a marked increase in the overall population of total macrophages (CD11b^+^ F4/80^+^), especially pro‐inflammatory tumor necrosis factor‐alpha (TNF‐α^+^) M1‐like macrophages (CD11b^+^ F4/80^+^ CD86^+^; Fig. 5D) in spleen, while the population of M2‐like macrophages (CD11b^+^ F4/80^+^ IL‐4R^+^ CD206^+^) declined significantly (Fig. 5E and Fig. S10).

In a similar fashion, we noted an enrichment of TNF‐α^+^ macrophages (Fig. S8C) in tumor homogenates, while the macrophage population expressing IL‐4R and CD206 were reduced (Fig. S8D). These results further suggest that DABIL‐4 targets and depletes IL‐4R^+^ MDSCs and M2‐like macrophages in both the TME and the spleen, thereby reducing the ability of these cells to suppress an antitumor immune response.

### 3.4 DABIL‐4 administration increases effector T‐cell populations and their cytotoxic potential *in vivo*

**Fig. 6:**
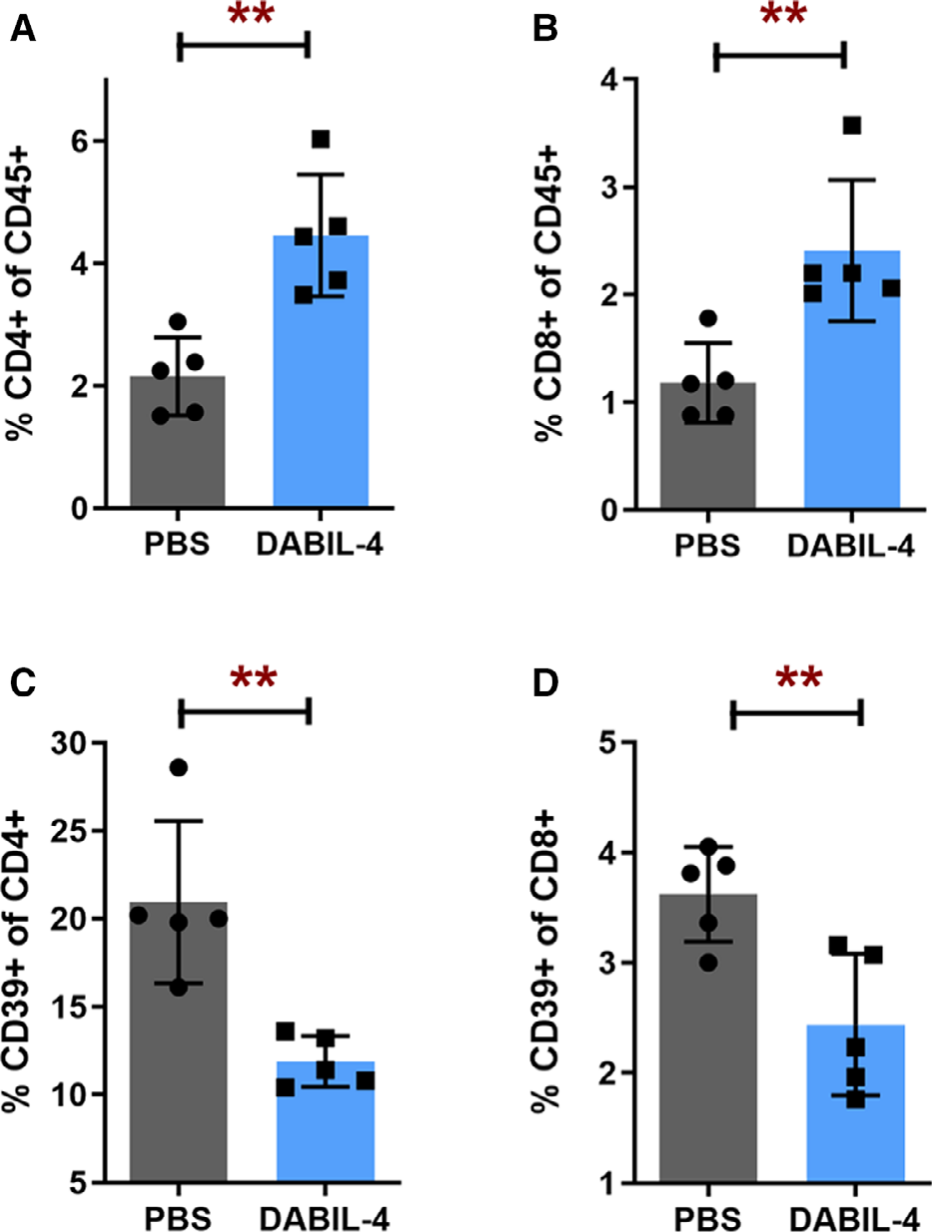
DABIL‐4 administration promotes the accumulation of lymphoid cell populations in spleen. Single‐cell suspensions of spleen were stained and analyzed by flow cytometry (*n* = 5). We evaluated the differences in the population of (A) CD4^+^ and (B) CD8^+^ T cells of CD45^+^ cells. We also noted the differences in (C) CD39 expression upon CD4^+^ and (D) CD8^+^ T cells. All panels correspond to day 25 post‐tumor implantation. Statistical significance between the groups was assessed by two‐tailed unpaired Student's *t*‐test considering an unequal distribution. Data are represented as mean ± SD. **P < 0.01.

Since the depletion of MDSCs and TAMs was anticipated to result in an increase in the Teff response to the 4T1 tumor, we investigated the effect of DABIL‐4 administration upon both CD4^+^ and CD8^+^ lymphocytes in 4T1 tumor‐bearing mice. As in Fig. 2A, mice began thrice‐weekly DABIL‐4 or PBS treatment on day 7 after 4T1 tumor implantation and homogenates from spleens and tumors were analyzed using multicolor flow cytometry. In spleens, we detected enrichment of CD4^+^ and CD8^+^ lymphocytes (Fig. 6A,B). We also observed ~ 70% reduction in the frequency of IL‐10‐producing CD4^+^ T cells (Fig. S11A) and a reduced expression of CD39 on both CD4^+^ and CD8^+^ lymphocyte subsets (Fig. 6C,D). CD39 cells along with CD73 are major ectonucleotidases that are known to scavenge pro‐inflammatory nucleotide mediators present in the TME and to generate immunosuppressive adenosine nucleosides that promote suppression of effector T cells and tumor growth [36, 37].

**Fig. 7:**
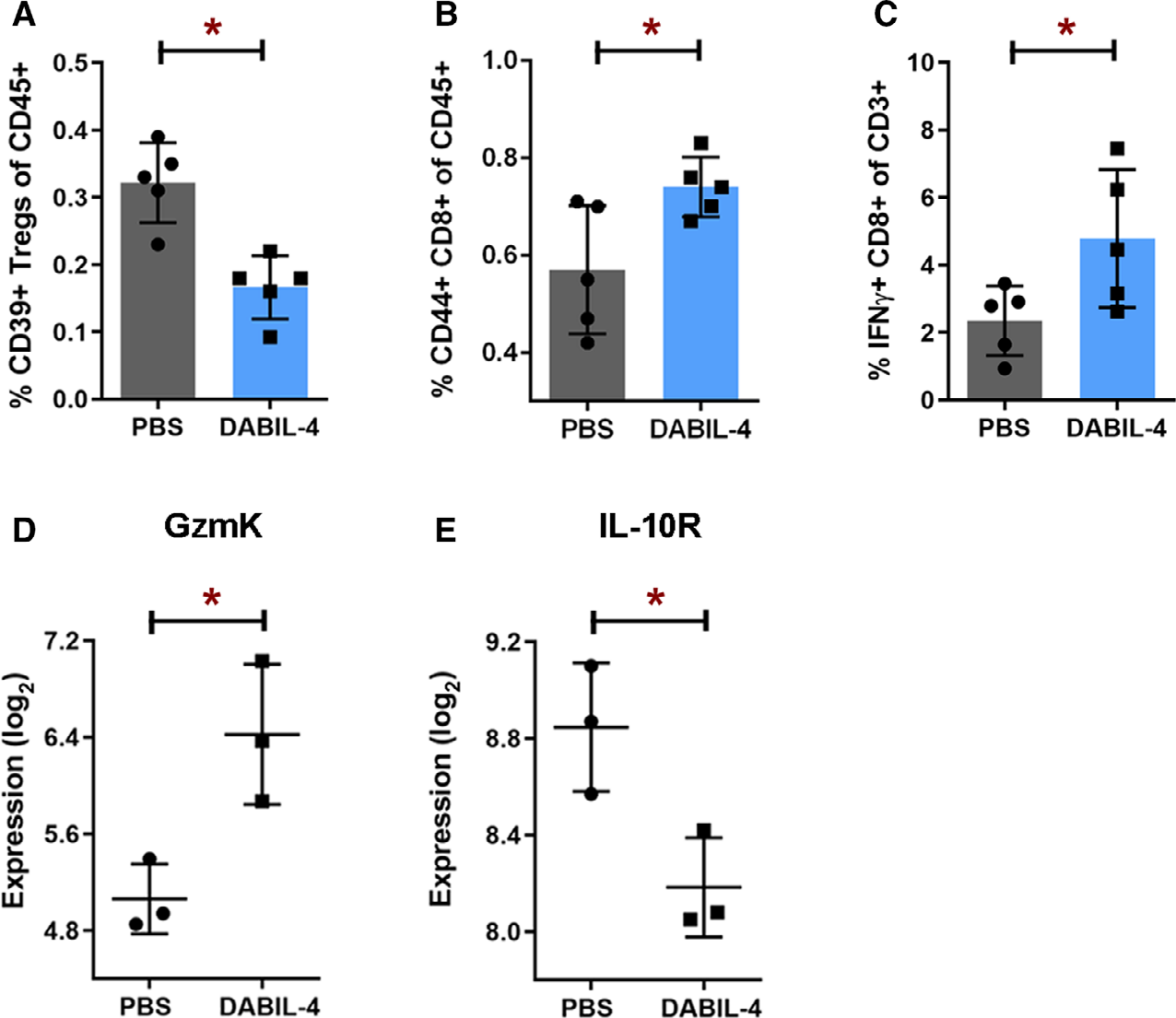
DABIL‐4 treatment promotes infiltration of effector cell populations in TME, mediating antitumor response. As in Fig. 2A, mice were treated with DABIL‐4 thrice weekly beginning on day 7, and they were sacrificed on days 17 and 25. Single‐cell suspensions of the tumors isolated from both the groups were stained and were analyzed by flow cytometry (*n* = 5). We found the differences in the population of (A) CD39^+^ Tregs of CD45^+^, (B) activated CD44^+^ CD8^+^ T cells of CD45^+^, and (C) IFN‐γ^+^ CD8^+^ T cells of CD3^+^ cells. Total RNA isolated from these tumors was subjected to NanoString PanCancer Immune Profiling Panel analysis and nSolver software (*n* = 3). Expression profiles of (D) GzmK and (E) IL‐10 receptor transcripts are shown for both treatment groups. RNA expression values are given as log_2_. All panels correspond to day 17 post‐tumor implantation except panel C that corresponds to day 25. All data are shown as mean ± SD. Statistical significance between the groups was assessed by two‐tailed unpaired Student's *t*‐test considering an unequal distribution. *P < 0.05  .

In the TME, DABIL‐4 treatment also depleted CD39^+^ activated Tregs (CD3^+^ CD4^+^ CD25^+^ FoxP3^+^ CD39^+^; Fig. 7A and Fig. S12). Murine Tregs overexpress CD39, further enhancing their ability to suppress effector T cells [38, 39]. Accordingly, the loss of Tregs was accompanied by an increased population of effector CD8^+^ T cells expressing CD44 and IFN‐γ in the TME (Fig. 7B,C). The change in the lymphocyte population was consistent with the elevated Granzyme K (GzmK) and decreased IL‐10 receptor mRNA transcript levels in the tumor (Fig. 7D,E and Table 1), which may indicate either enhanced cytotoxic potential of lymphocytes or an overall increase in lymphocyte population in the TME.

We also evaluated the impact of DABIL‐4 administration upon IL‐4R^+^ natural killer (NK; CD49b^+^) and B lymphocytes (CD19^+^). While we did not observe significant differences in the population of IL‐4R^+^ NK cells in the spleen of DABIL‐4‐treated mice (Fig. S11B), we did observe a marked decrease in the overall population of IL‐4R^+^ B cells (Fig. S11C) and regulatory B lymphocytes (Bregs) also known as IL‐10‐producing B cells (Fig. S11D). Bregs are the immunosuppressive lymphocytes, which have been shown to promote breast cancer metastasis by inducing the conversion of resting CD4^+^ T cells into Tregs [40].

Taken together, based on these observations we infer that DABIL‐4‐mediated depletion of immunosuppressive cell populations facilitates significant increases in the population of activated effector T cells in the TME.

## 4 Discussion

Here, we report that the DT‐based fusion protein, DABIL‐4, is a highly potent and selective cytotoxic agent that targets TNBC cells along with the immunosuppressive myeloid cell populations expressing IL‐4R. The fusion protein toxin inhibits tumor growth and markedly reduces lung metastases in a murine syngeneic TNBC adenocarcinoma model. The primary mode of DABIL‐4 action is the depletion of IL‐4R^+^ immunosuppressive PMN‐MDSCs and M2‐like macrophages, thereby facilitating a more robust antitumor T‐cell response. Almost 30 years ago, Lakkis *et al*. [11] reported a similar IL‐4R^+^ DT‐based fusion protein that was expressed from recombinant *E. coli* and demonstrated targeted cytotoxicity toward IL‐4R‐bearing eukaryotic cells *in vitro* and a marked reduction in delayed‐type hypersensitivity *in vivo*. Since this has been the only prior report describing DABIL‐4, the potential therapeutic efficacy of this fusion protein was not evaluated in preclinical animal tumor models and a more detailed understanding of its action against host immune cells remained unknown. A similar immunotoxin, IL‐4(38–37)‐PE38KDEL, comprised of a circularly permutated human IL‐4 fused with *Pseudomonas aeruginosa* exotoxin A, was found to be cytotoxic against the human breast cancer cell line, MDA‐MB231, both *in vitro* and in murine xenograft models [41]. It was also tested in preclinical models of various human malignancies including breast cancer [2, 41]. These studies with IL‐4(38–37)‐PE38KDEL were carried out in immunocompromised mice also without an assessment of its effect on IL‐4R^+^ immune cells [41]. As a result, the effect of this fusion protein toxin upon the host immune system remained undeciphered. While several studies have shown therapeutic benefit of IL‐4R targeting [4, 42], the present study is the first to demonstrate the immunomodulatory activity of an IL‐4‐targeted diphtheria fusion toxin.

In the present study, we demonstrate that DABIL‐4 has not only a potent and direct cytotoxic activity against IL‐4R‐positive TNBC cells, but it also targets PMN‐MDSCs and M2‐like macrophages *in vivo*. In order to assess the relative degree of direct 4T1 tumor cell killing by DABIL‐4, we established 4T1 TNBC tumors in the mammary fat pads of IL‐4R KO mice and then treated these animals with DABIL‐4. In this model, we found only modest reductions in tumor progression (Fig. 5B–D). While we did not test DABIL‐4 tumors against IL‐4R‐negative tumors, these results strongly suggest that the depletion of PMN‐MDSCs and M2‐like macrophages in 4T1 tumor‐bearing mice accounts for a significant degree of DABIL‐4 antitumor efficacy. This antitumor effect is likely further enhanced by a concomitant reduction in Tregs and the concomitant increase in effector T‐cell populations in the TME. As further evidence of immunomodulatory activity of the fusion toxin in the context of infectious diseases, we recently demonstrated that DABIL‐4 administration depletes MDSCs and M2 macrophages leading to the better bacterial clearance in *Mycobacterium tuberculosis*‐infected mice lungs [43]. Hence, the data from IL‐4R KO mice and the data from tuberculosis strongly suggest that both direct tumor killing and an adjuvant immunomodulatory activity are responsible for the DABIL‐4‐mediated therapeutic benefit.

In addition, we observed a selective depletion of splenic PMN‐MDSCs, while the population of splenic M‐MDSCs remained the same in DABIL‐4‐treated mice. Interestingly, in spleens of 4T1 tumor‐bearing mice, PMN‐MDSCs constituted the major MDSC subset and exhibited higher proliferation rate compared with M‐MDSCs [44, 45]. This higher rate of proliferation could possibly be responsible for the increased susceptibility of PMN‐MDSCs to DABIL‐4, which catalyzes the NAD^+^‐dependent ADP‐ribosylation of elongation factor 2 and thereby blocks protein synthesis [46]. In many cancers, M‐MDSCs have been shown to be abundant in the TME and tend to rapidly differentiate into TAMs [44]. However, considering the limited ability to distinguish M‐MDSCs from macrophages [47], the observed depletion of TAMs may also arise from targeting of both TAMs and M‐MDSCs.

In 4T1 tumor‐bearing mice, the spleen is known to be the primary site of MDSC proliferation and sequestration [30, 45], and the site from which these cells are subsequently recruited to the TME in response to tumor‐secreted chemokines [48]. Accordingly, splenectomy has been shown to reduce the amount of tumor‐infiltrating MDSC and to cause tumor regression in murine cancer models [49], suggesting that splenic MDSCs may also contribute to tumorigenesis [50]. Indeed, this observation may at least partially explain why DABIL‐4‐mediated depletion of splenic MDSCs results in a robust antitumor response. Despite this robust response and an *in vitro* IC_50_ of 2–4 nm, DABIL‐4 treatment of mice led to incomplete tumor eradication. Reasons for this may be the aggressiveness of tumors in the 4T1 model, poor drug penetration into tumors, and the short half‐life of DT fusion proteins (~ 90 min) such as DABIL‐4. Our future work will focus on approaches to improve drug exposure to the tumors, and also testing DABIL‐4 in combination with other agents in preclinical models. Additionally, as the mouse IL‐4R/IL‐4 interaction is species‐specific in that mouse IL‐4 does cross‐react with human IL‐4R, we were unable to perform cytotoxicity assays with DABIL‐4, either against human breast cancer cell lines *in vitro* or with *in vivo* murine models of human breast cancer. We are currently focusing our efforts on the generation of humanized DABIL‐4 for future studies. In summary, DABIL‐4 is a promising therapeutic that demonstrates both direct killing of IL‐4R‐positive tumor cells combined with the depletion of IL‐4R‐bearing immunosuppressive cells such as MDSCs and TAMs. As such, it holds potential as dual cytotoxic and immunotherapeutic agent for the treatment of multiple cancers including TNBC and warrants further study.

## 5 Conclusions

In summary, solid tumors such as breast cancer express IL‐4R on both tumor and immunosuppressive cells in the TME. In this study, we have utilized a fusion protein toxin, DABIL‐4, composed of the catalytic and translocation domains of DT and murine IL‐4 in order to selectively target and eliminate both of these cell populations. We demonstrate that DABIL‐4 specifically eliminates 4T1 TNBC cells *in vitro*. *In vivo* DABIL‐4 markedly inhibited 4T1 tumor growth and metastasis to the lung; however, the primary activity of this fusion protein toxin was found to be the depletion of IL‐4R^+^ MDSCs, TAM, and Tregs.

## Conflict of interest

JRM and WRB hold positions in Sonoval, LLC, which holds rights to develop certain DT‐based fusion proteins.

### Peer Review

The peer review history for this article is available at https://publons.com/publon/10.1002/1878‐0261.12938.

## Author contributions

SP, JRM, DS, and WRB conceptualized the study and designed the research approach; SP, SS, AK, and JS performed research; LSC and JRM contributed new reagents/analytic tools; SP, JRM, DS, and WRB analyzed the data; and SP, JRM, DS, and WRB wrote the paper, and SP, SS, LSC, AK, JS, JRM, DS, and WRB critically reviewed the manuscript.

## Supporting information


**Fig. S1.** pKN2.6Z‐LC128 shuttle vector plasmid map.

**Fig. S2.** Genetic construction and purification of DABIL‐4 fusion toxin using *C. diphtheriae*.

**Fig. S3.** Cytotoxic activity of DABIL‐4 against NT2.5 cells tested using MTS‐based assay

**Fig. S4.** DABIL‐4 induces apoptosis in IL‐4R^+^ tumor cells.

**Fig. S5.** DABIL‐4 administration induces apoptosis in 4T1 tumors *in vivo*.

**Fig. S6.** DABIL‐4 exhibits anti‐tumor activity in E0771 adenocarcinoma model in C57BL/6 mice.

**Fig. S7.** Quantification of lung metastases using IVIS bioluminescence imaging.

**Fig. S8.** DABIL‐4 treatment modulates myeloid cell populations in spleen and tumor microenvironment.

**Fig. S9.** Two‐dimensional representation of FACS data to demonstrate depletion of IL‐4R^+^ PMN‐MDSCs in spleen isolated from DABIL‐4 treated mice on day 25.

**Fig. S10.** Two‐dimensional representation of FACS data to show depletion of M2 macrophages in spleens isolated from DABIL‐4 treated mice on day 25.

**Fig. S11.** DABIL‐4 administration depletes IL‐4R^+^ B‐cells, Bregs and IL‐10^+^ CD4^+^ T‐cell populations in spleen.

**Fig. S12.** Two‐dimensional representation of FACS data to show depletion of CD39^+^ Tregs in tumors isolated from DABIL‐4 treated mice on day 17.

Supplementary Material

## Data Availability

The data that support the findings of this study are available in main texts (Table 1 and Figs 1, 2, 3, 4, 5, 6, 7) and the supplementary material (Figs S1–S12) of the manuscript.

## References

[1] Setrerrahmane S & Xu H (2017) Tumor‐related interleukins: old validated targets for new anti‐cancer drug development. Mol Cancer 16, 153. 28927416 10.1186/s12943-017-0721-9 PMC5606116

[2] Suzuki A , Leland P , Joshi BH & Puri RK (2015) Targeting of IL‐4 and IL‐13 receptors for cancer therapy. Cytokine 75, 79–88. 26088753 10.1016/j.cyto.2015.05.026

[3] Bankaitis KV & Fingleton B (2015) Targeting IL4/IL4R for the treatment of epithelial cancer metastasis. Clin Exp Metastasis 32, 847–856. 26385103 10.1007/s10585-015-9747-9 PMC4651701

[4] Gaggianesi M , Turdo A , Chinnici A , Lipari E , Apuzzo T , Benfante A , Sperduti I , Di Franco S , Meraviglia S , Lo Presti E *et al*. (2017) IL4 primes the dynamics of breast cancer progression via DUSP4 inhibition. Cancer Res 77, 3268–3279. 28400477 10.1158/0008-5472.CAN-16-3126

[5] Konig A , Vilsmaier T , Rack B , Friese K , Janni W , Jeschke U , Andergassen U , Trapp E , Juckstock J , Jager B *et al*. (2016) Determination of interleukin‐4, ‐5, ‐6, ‐8 and ‐13 in serum of patients with breast cancer before treatment and its correlation to circulating tumor cells. Anticancer Res 36, 3123–3130. 27272837

[6] Venmar KT , Carter KJ , Hwang DG , Dozier EA & Fingleton B (2014) IL4 receptor ILR4alpha regulates metastatic colonization by mammary tumors through multiple signaling pathways. Cancer Res 74, 4329–4340. 24947041 10.1158/0008-5472.CAN-14-0093 PMC4134711

[7] Venmar KT , Kimmel DW , Cliffel DE & Fingleton B (2015) IL4 receptor alpha mediates enhanced glucose and glutamine metabolism to support breast cancer growth. Biochim Biophys Acta 1853, 1219–1228. 25746764 10.1016/j.bbamcr.2015.02.020 PMC4380623

[8] Lyons TG (2019) Targeted therapies for triple‐negative breast cancer. Curr Treat Options Oncol 20, 82. 31754897 10.1007/s11864-019-0682-x

[9] Lyons TG & Traina TA (2019) Emerging novel therapeutics in triple‐negative breast cancer. Adv Exp Med Biol 1152, 377–399. 31456195 10.1007/978-3-030-20301-6_20

[10] Mina LA , Lim S , Bahadur SW & Firoz AT (2019) Immunotherapy for the treatment of breast cancer: emerging new data. Breast Cancer 11, 321–328. 32099454 10.2147/BCTT.S184710 PMC6997226

[11] Lakkis F , Steele A , Pacheco‐Silva A , Rubin‐Kelley V , Strom TB & Murphy JR (1991) Interleukin 4 receptor targeted cytotoxicity: genetic construction and *in vivo* immunosuppressive activity of a diphtheria toxin‐related murine interleukin 4 fusion protein. Eur J Immunol 21, 2253–2258. 1679715 10.1002/eji.1830210937

[12] Cheung LS , Fu J , Kumar P , Kumar A , Urbanowski ME , Ihms EA , Parveen S , Bullen CK , Patrick GJ , Harrison R *et al*. (2019) Second‐generation IL‐2 receptor‐targeted diphtheria fusion toxin exhibits antitumor activity and synergy with anti‐PD‐1 in melanoma. Proc Natl Acad Sci USA 116, 3100–3105. 30718426 10.1073/pnas.1815087116 PMC6386727

[13] Kim JB , Urban K , Cochran E , Lee S , Ang A , Rice B , Bata A , Campbell K , Coffee R , Gorodinsky A *et al*. (2010) Non‐invasive detection of a small number of bioluminescent cancer cells *in vivo* . PLoS One 5, e9364. 20186331 10.1371/journal.pone.0009364 PMC2826408

[14] Johnstone CN , Smith YE , Cao Y , Burrows AD , Cross RS , Ling X , Redvers RP , Doherty JP , Eckhardt BL , Natoli AL *et al*. (2015) Functional and molecular characterisation of EO771.LMB tumours, a new C57BL/6‐mouse‐derived model of spontaneously metastatic mammary cancer. Dis Model Mech 8, 237–251. 25633981 10.1242/dmm.017830 PMC4348562

[15] Le Naour A , Rossary A & Vasson M‐P (2020) EO771, is it a well‐characterized cell line for mouse mammary cancer model? Limit and uncertainty. Cancer Med 9, 8074–8085. 33026171 10.1002/cam4.3295 PMC7643677

[16] Reilly RT , Gottlieb MB , Ercolini AM , Machiels JP , Kane CE , Okoye FI , Muller WJ , Dixon KH & Jaffee EM (2000) HER‐2/neu is a tumor rejection target in tolerized HER‐2/neu transgenic mice. Cancer Rese 60, 3569–3576. 10910070

[17] Ratts R , Trujillo C , Bharti A , vanderSpek J , Harrison R & Murphy JR (2005) A conserved motif in transmembrane helix 1 of diphtheria toxin mediates catalytic domain delivery to the cytosol. Proc Natl Acad Sci USA 102, 15635–15640. 16230620 10.1073/pnas.0504937102 PMC1257389

[18] Trujillo C , Taylor‐Parker J , Harrison R & Murphy JR (2010) Essential lysine residues within transmembrane helix 1 of diphtheria toxin facilitate COPI binding and catalytic domain entry. Mol Microbiol 76, 1010–1019. 20398220 10.1111/j.1365-2958.2010.07159.x PMC4113555

[19] Yamaizumi M , Mekada E , Uchida T & Okada Y (1978) One molecule of diphtheria toxin fragment A introduced into a cell can kill the cell. Cell 15, 245–250. 699044 10.1016/0092-8674(78)90099-5

[20] Pulaski BA & Ostrand‐Rosenberg S (2001) Mouse 4T1 breast tumor model. Curr Protoc Immunol Chapter 20, Unit 20.22. 10.1002/0471142735.im2002s39 18432775

[21] Pascual G , Avgustinova A , Mejetta S , Martin M , Castellanos A , Attolini CS , Berenguer A , Prats N , Toll A , Hueto JA *et al*. (2017) Targeting metastasis‐initiating cells through the fatty acid receptor CD36. Nature 541, 41–45. 27974793 10.1038/nature20791

[22] Chien MH , Lee WJ , Yang YC , Li YL , Chen BR , Cheng TY , Yang PW , Wang MY , Jan YH , Lin YK *et al*. (2017) KSRP suppresses cell invasion and metastasis through miR‐23a‐mediated EGR3 mRNA degradation in non‐small cell lung cancer. Biochim Biophys Acta Gene Regul Mech 1860, 1013–1024. 28847731 10.1016/j.bbagrm.2017.08.005

[23] Pio R , Jia Z , Baron VT & Mercola D (2013) Early growth response 3 (Egr3) is highly over‐expressed in non‐relapsing prostate cancer but not in relapsing prostate cancer. PLoS One 8, e54096. 23342084 10.1371/journal.pone.0054096 PMC3544741

[24] Suzuki T , Inoue A , Miki Y , Moriya T , Akahira J , Ishida T , Hirakawa H , Yamaguchi Y , Hayashi S & Sasano H (2007) Early growth responsive gene 3 in human breast carcinoma: a regulator of estrogen‐meditated invasion and a potent prognostic factor. Endocr Relat Cancer 14, 279–292. 17639044 10.1677/ERC-06-0005

[25] Pavlovic M , Arnal‐Estape A , Rojo F , Bellmunt A , Tarragona M , Guiu M , Planet E , Garcia‐Albeniz X , Morales M , Urosevic J *et al*. (2015) Enhanced MAF oncogene expression and breast cancer bone metastasis. J Natl Cancer Inst 107, djv256. 26376684 10.1093/jnci/djv256 PMC4681582

[26] Yao M , Fang W , Smart C , Hu Q , Huang S , Alvarez N , Fields P & Cheng N (2019) CCR2 chemokine receptors enhance growth and cell‐cycle progression of breast cancer cells through SRC and PKC activation. Mol Cancer Res 17, 604–617. 30446625 10.1158/1541-7786.MCR-18-0750 PMC6359961

[27] Boyle ST , Faulkner JW , McColl SR & Kochetkova M (2015) The chemokine receptor CCR6 facilitates the onset of mammary neoplasia in the MMTV‐PyMT mouse model via recruitment of tumor‐promoting macrophages. Mol Cancer 14, 115. 26047945 10.1186/s12943-015-0394-1 PMC4464622

[28] Erin N , Podnos A , Tanriover G , Duymus O , Cote E , Khatri I & Gorczynski RM (2015) Bidirectional effect of CD200 on breast cancer development and metastasis, with ultimate outcome determined by tumor aggressiveness and a cancer‐induced inflammatory response. Oncogene 34, 3860–3870. 25263452 10.1038/onc.2014.317

[29] Talebian F , Liu JQ , Liu Z , Khattabi M , He Y , Ganju R & Bai XF (2012) Melanoma cell expression of CD200 inhibits tumor formation and lung metastasis via inhibition of myeloid cell functions. PLoS One 7, e31442. 22319630 10.1371/journal.pone.0031442 PMC3272017

[30] DuPre SA & Hunter KW Jr (2007) Murine mammary carcinoma 4T1 induces a leukemoid reaction with splenomegaly: association with tumor‐derived growth factors. Exp Mol Pathol 82, 12–24. 16919266 10.1016/j.yexmp.2006.06.007

[31] Dysthe M & Parihar R (2020) Myeloid‐derived suppressor cells in the tumor microenvironment. Adv Exp Med Biol 1224, 117–140. 32036608 10.1007/978-3-030-35723-8_8

[32] Nakamura K & Smyth MJ (2020) Myeloid immunosuppression and immune checkpoints in the tumor microenvironment. Cell Mol Immunol 17, 1–12. 31611651 10.1038/s41423-019-0306-1 PMC6952382

[33] Veglia F , Perego M & Gabrilovich D (2018) Myeloid‐derived suppressor cells coming of age. Nat Immunol 19, 108–119. 29348500 10.1038/s41590-017-0022-x PMC5854158

[34] Wang HW & Joyce JA (2010) Alternative activation of tumor‐associated macrophages by IL‐4: priming for protumoral functions. Cell Cycle 9, 4824–4835. 21150330 10.4161/cc.9.24.14322 PMC3047808

[35] Ostrand‐Rosenberg S & Sinha P (2009) Myeloid‐derived suppressor cells: linking inflammation and cancer. J Immunol (Baltimore, Md : 1950) 182, 4499–4506, 10.4049/jimmunol.0802740 PMC2810498 19342621

[36] Allard B , Longhi MS , Robson SC & Stagg J (2017) The ectonucleotidases CD39 and CD73: Novel checkpoint inhibitor targets. Immunol Rev 276, 121–144. 28258700 10.1111/imr.12528 PMC5338647

[37] Canale FP , Ramello MC , Núñez N , Furlan CLA , Bossio SN , Serrán MG , Boari JT , del Castillo A , Ledesma M , Sedlik C *et al*. (2018) CD39 expression defines cell exhaustion in tumor‐infiltrating CD8^+^ T cells. Cancer Res 78, 115–128. 29066514 10.1158/0008-5472.CAN-16-2684

[38] Deaglio S , Dwyer KM , Gao W , Friedman D , Usheva A , Erat A , Chen JF , Enjyoji K , Linden J , Oukka M *et al*. (2007) Adenosine generation catalyzed by CD39 and CD73 expressed on regulatory T cells mediates immune suppression. J Exp Med 204, 1257–1265. 17502665 10.1084/jem.20062512 PMC2118603

[39] Ohta A & Sitkovsky M (2014) Extracellular adenosine‐mediated modulation of regulatory T cells. Front Immunol 5, 304. 25071765 10.3389/fimmu.2014.00304 PMC4091046

[40] Olkhanud PB , Damdinsuren B , Bodogai M , Gress RE , Sen R , Wejksza K , Malchinkhuu E , Wersto RP & Biragyn A (2011) Tumor‐evoked regulatory B cells promote breast cancer metastasis by converting resting CD4^+^ T cells to T‐regulatory cells. Cancer Res 71, 3505–3515. 21444674 10.1158/0008-5472.CAN-10-4316 PMC3096701

[41] Leland P , Taguchi J , Husain SR , Kreitman RJ , Pastan I & Puri RK (2000) Human breast carcinoma cells express type II IL‐4 receptors and are sensitive to antitumor activity of a chimeric IL‐4‐pseudomonas exotoxin fusion protein *in vitro* and *in vivo* . Mol Med 6, 165–178. 10965493 PMC1949945

[42] Roth F , De La Fuente AC , Vella JL , Zoso A , Inverardi L & Serafini P (2012) Aptamer‐mediated blockade of IL4Ralpha triggers apoptosis of MDSCs and limits tumor progression. Cancer Res 72, 1373–1383. 22282665 10.1158/0008-5472.CAN-11-2772

[43] Parveen S , Lun S , Urbanowski ME , Cardin M , Murphy JR & Bishai WR (2020) Effective host‐directed therapy for tuberculosis by targeted depletion of myeloid‐derived suppressor cells using a diphtheria toxin‐based fusion protein. bioRxiv [PREPRINT]. 10.1093/infdis/jiab235 PMC8643419 33955457

[44] Kumar V , Patel S , Tcyganov E & Gabrilovich DI (2016) The nature of myeloid‐derived suppressor cells in the tumor microenvironment. Trends Immunol 37, 208–220. 26858199 10.1016/j.it.2016.01.004 PMC4775398

[45] Younos IH , Dafferner AJ , Gulen D , Britton HC & Talmadge JE (2012) Tumor regulation of myeloid‐derived suppressor cell proliferation and trafficking. Int Immunopharmacol 13, 245–256. 22609473 10.1016/j.intimp.2012.05.002

[46] Warner JR (1999) The economics of ribosome biosynthesis in yeast. Trends Biochem Sci 24, 437–440. 10542411 10.1016/s0968-0004(99)01460-7

[47] Bronte V , Brandau S , Chen SH , Colombo MP , Frey AB , Greten TF , Mandruzzato S , Murray PJ , Ochoa A , Ostrand‐Rosenberg S *et al*. (2016) Recommendations for myeloid‐derived suppressor cell nomenclature and characterization standards. Nat Commun 7, 12150. 27381735 10.1038/ncomms12150 PMC4935811

[48] Yang L , Huang J , Ren X , Gorska AE , Chytil A , Aakre M , Carbone DP , Matrisian LM , Richmond A , Lin PC *et al*. (2008) Abrogation of TGF beta signaling in mammary carcinomas recruits Gr‐1+CD11b+ myeloid cells that promote metastasis. Cancer Cell 13, 23–35. 18167337 10.1016/j.ccr.2007.12.004 PMC2245859

[49] Mabuchi S , Matsumoto Y , Kawano M , Minami K , Seo Y , Sasano T , Takahashi R , Kuroda H , Hisamatsu T , Kakigano A *et al*. (2014) Uterine cervical cancer displaying tumor‐related leukocytosis: a distinct clinical entity with radioresistant feature. J Natl Cancer Inst 106, dju147. 24948742 10.1093/jnci/dju147

[50] Di Mitri D , Toso A & Alimonti A (2015) Molecular pathways: targeting tumor‐infiltrating myeloid‐derived suppressor cells for cancer therapy. Clin Cancer Res 21, 3108–3112. 25967145 10.1158/1078-0432.CCR-14-2261

[51] Inoue A , Omoto Y , Yamaguchi Y , Kiyama R & Hayashi SI (2004) Transcription factor EGR3 is involved in the estrogen‐signaling pathway in breast cancer cells. J Mol Endocrinol 32, 649–661. 15171706 10.1677/jme.0.0320649

[52] Bisgin A , Meng WJ , Adell G & Sun XF (2019) Interaction of CD200 overexpression on tumor cells with CD200R1 overexpression on stromal cells: an escape from the host immune response in rectal cancer patients. J Oncol 2019, 5689464. 30800162 10.1155/2019/5689464 PMC6360612

[53] Shresta S , Goda P , Wesselschmidt R & Ley TJ (1997) Residual cytotoxicity and granzyme K expression in granzyme A‐deficient cytotoxic lymphocytes. J Biol Chem 272, 20236–20244. 9242702 10.1074/jbc.272.32.20236

[54] Asadullah K , Sterry W & Volk HD (2003) Interleukin‐10 therapy–review of a new approach. Pharmacol Rev 55, 241–269. 12773629 10.1124/pr.55.2.4

