# Supplementary figures and images for: Therapeutic targeting with DABIL‐4 depletes myeloid suppressor cells in 4T1 triple‐negative breast cancer model
Source: Mol Oncol. 2021 Mar 24;15(5):1330–44. doi: 10.1002/1878-0261.12938 (PMC8096791; doi:10.1002/1878-0261.12938)

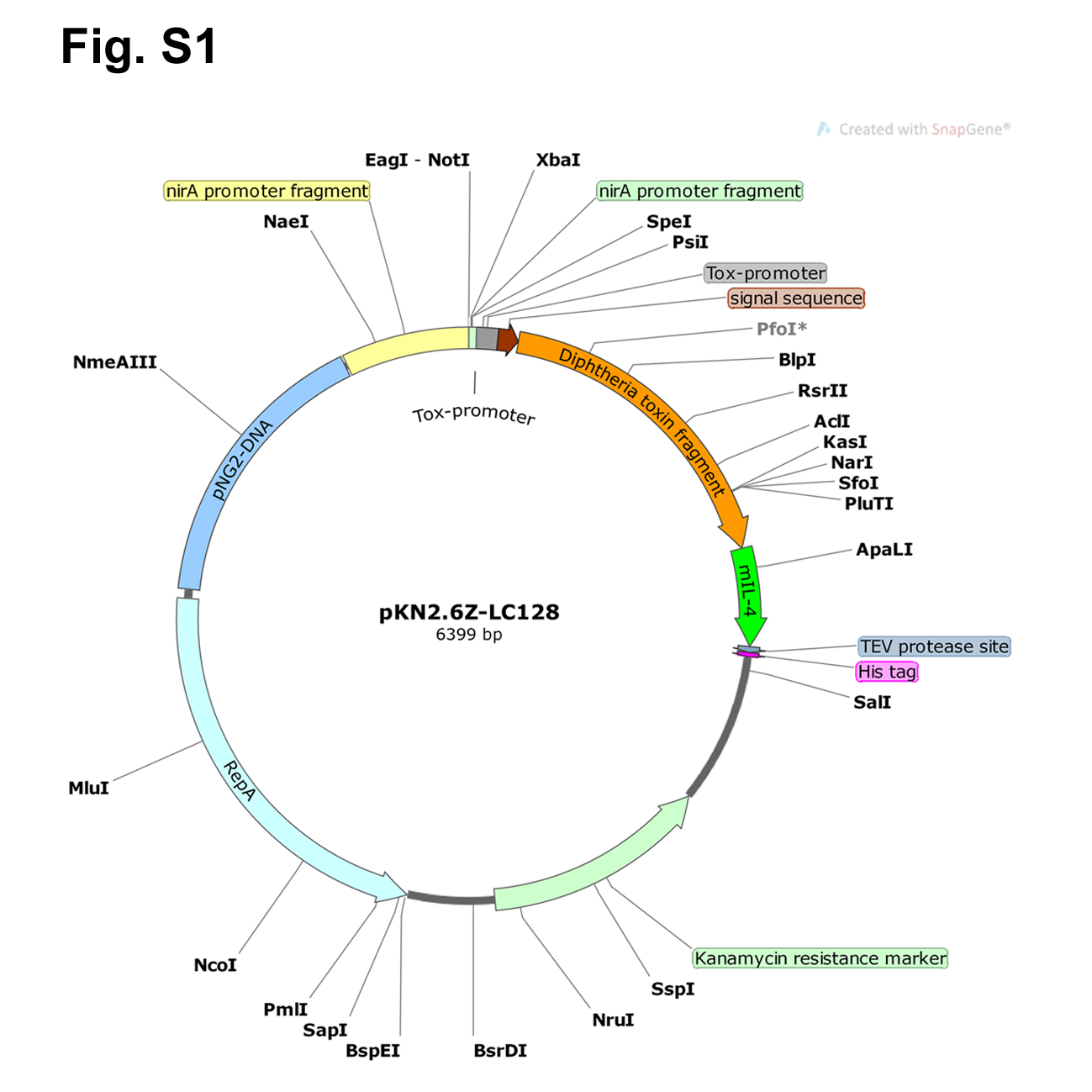

Supplement: Supplementary file 1 — Fig. S1. pKN2.6Z‐LC128 shuttle vector plasmid map. [file MOL2-15-1330-s005.tif]

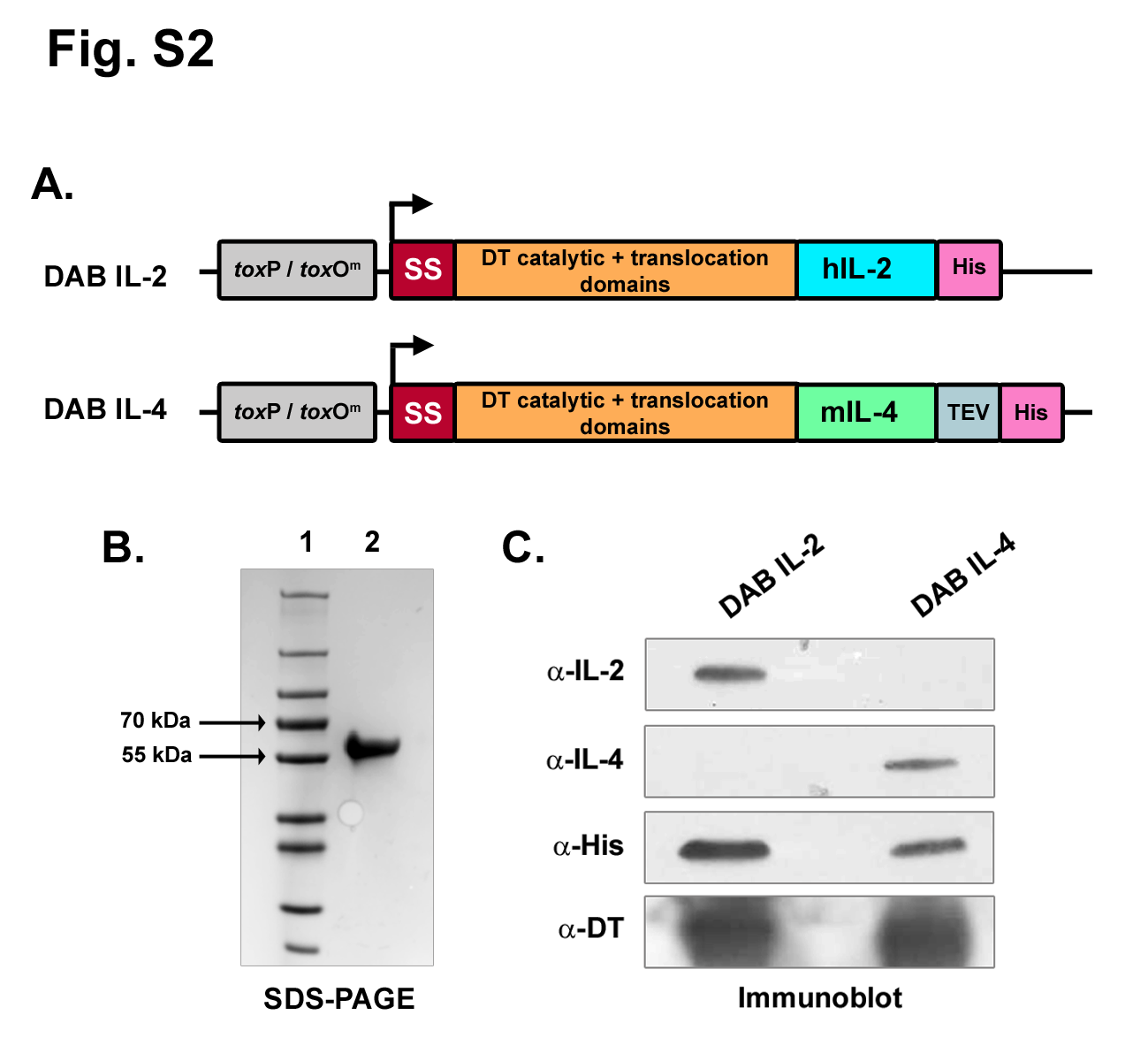

Supplement: Supplementary file 2 — Fig. S2. Genetic construction and purification of DABIL‐4 fusion toxin using C. diphtheriae. [file MOL2-15-1330-s002.tif]

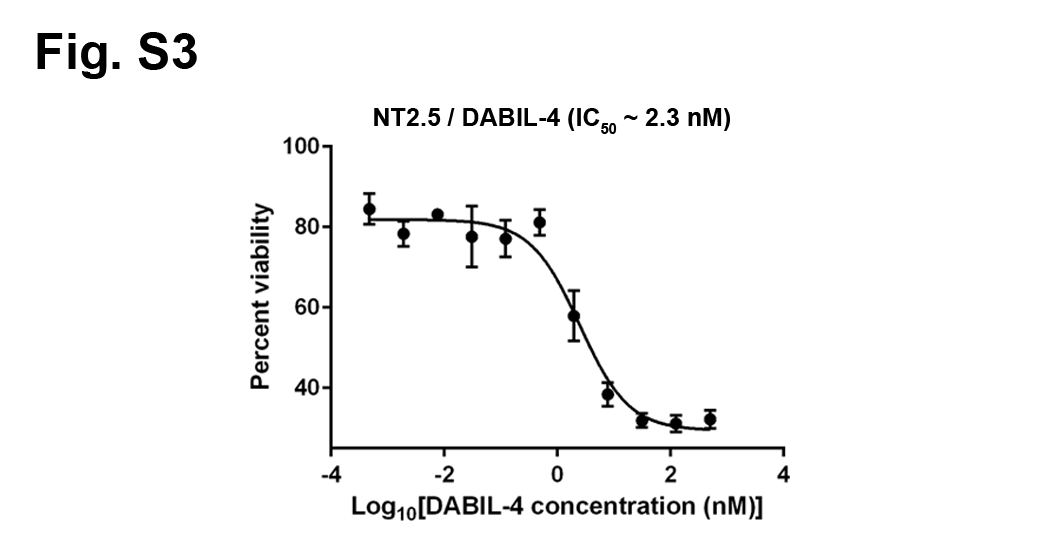

Supplement: Supplementary file 3 — Fig. S3. Cytotoxic activity of DABIL‐4 against NT2.5 cells tested using MTS‐based assay [file MOL2-15-1330-s004.tif]

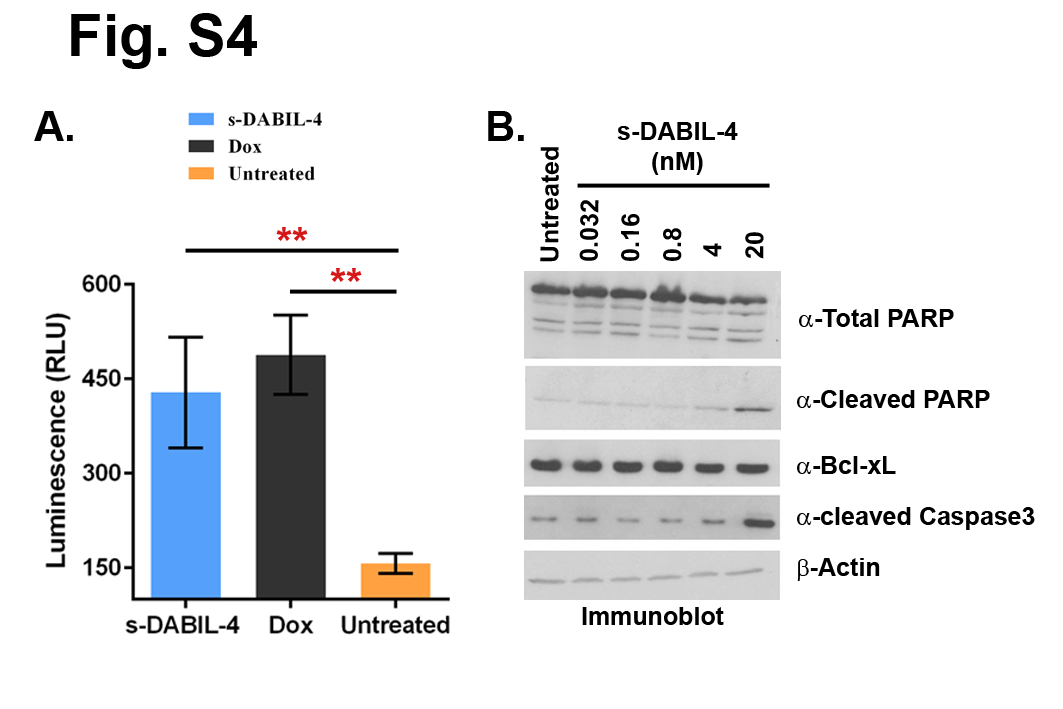

Supplement: Supplementary file 4 — Fig. S4. DABIL‐4 induces apoptosis in IL‐4R+ tumor cells. [file MOL2-15-1330-s001.tif]

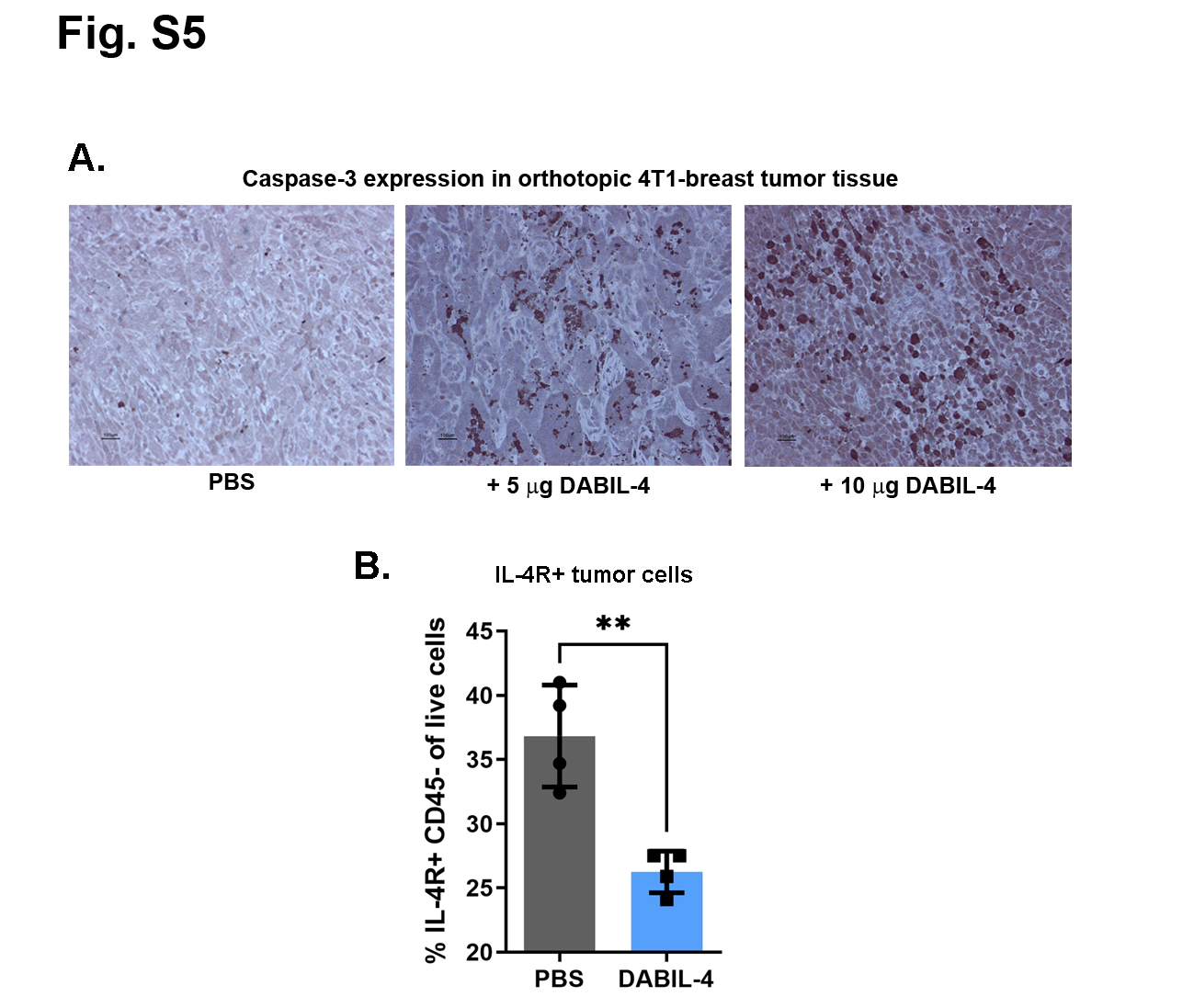

Supplement: Supplementary file 5 — Fig. S5. DABIL‐4 administration induces apoptosis in 4T1 tumors in vivo. [file MOL2-15-1330-s012.tif]

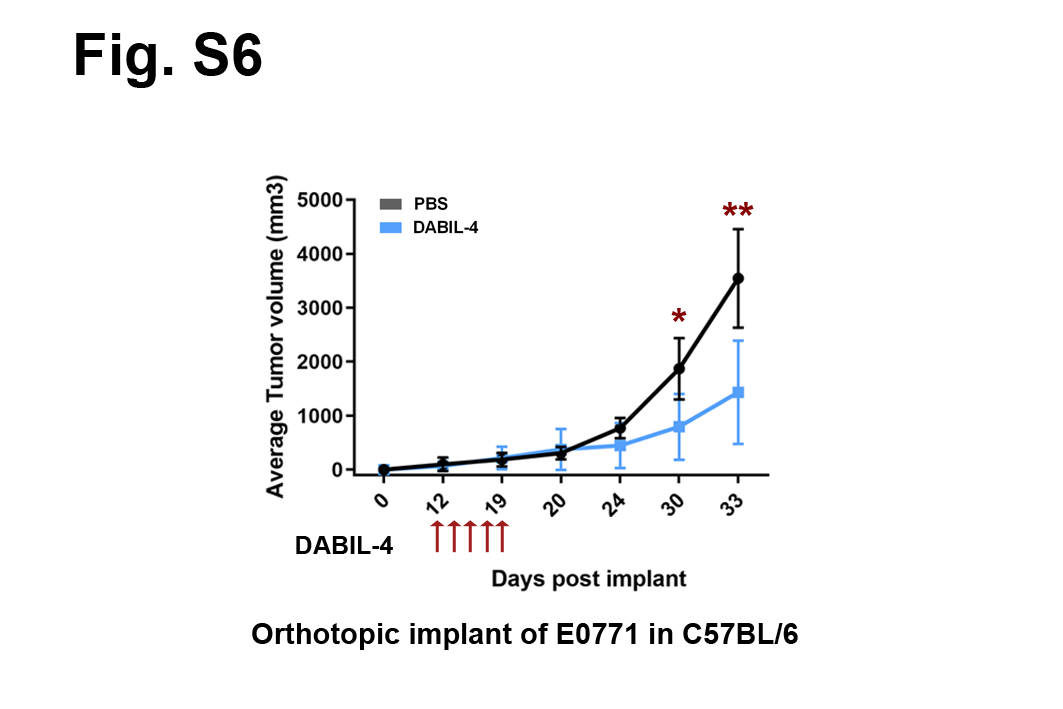

Supplement: Supplementary file 6 — Fig. S6. DABIL‐4 exhibits anti‐tumor activity in E0771 adenocarcinoma model in C57BL/6 mice. [file MOL2-15-1330-s008.tif]

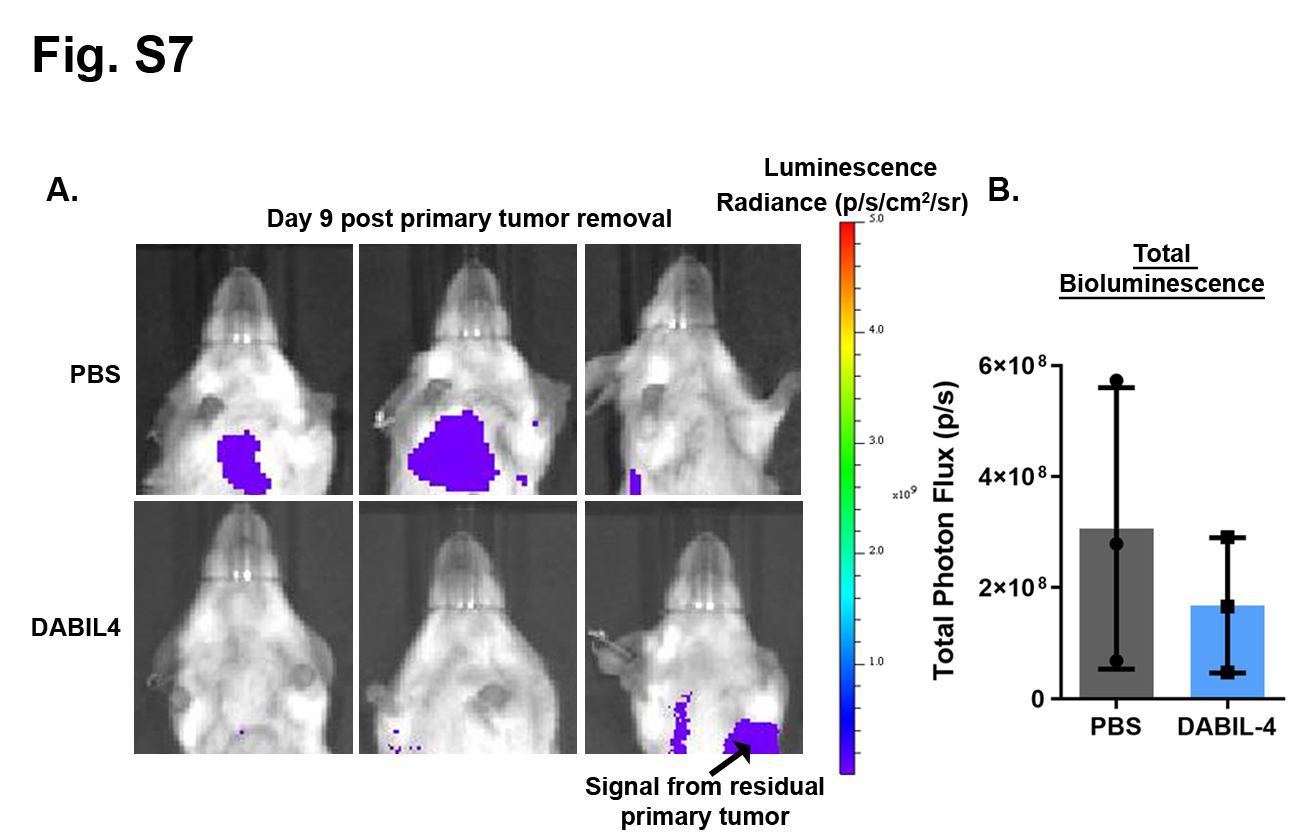

Supplement: Supplementary file 7 — Fig. S7. Quantification of lung metastases using IVIS bioluminescence imaging. [file MOL2-15-1330-s010.tif]

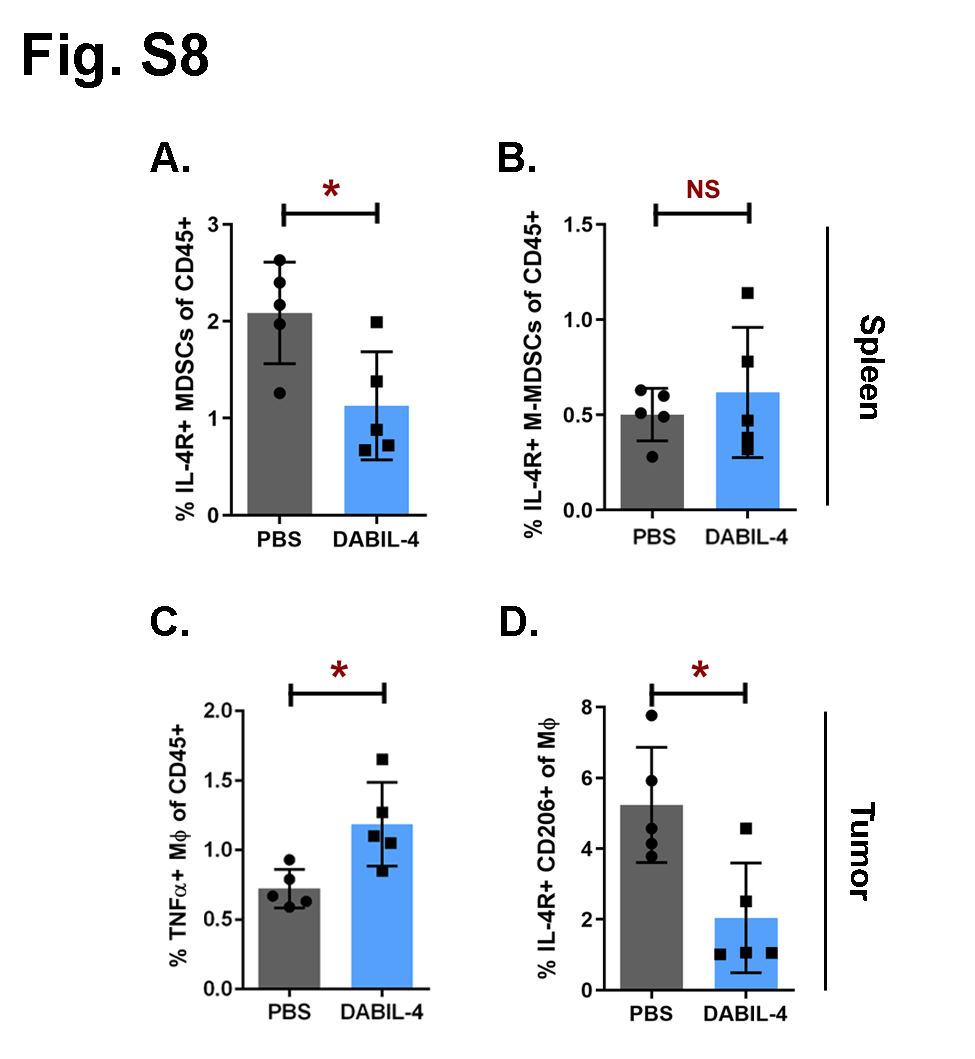

Supplement: Supplementary file 8 — Fig. S8. DABIL‐4 treatment modulates myeloid cell populations in spleen and tumor microenvironment. [file MOL2-15-1330-s011.tif]

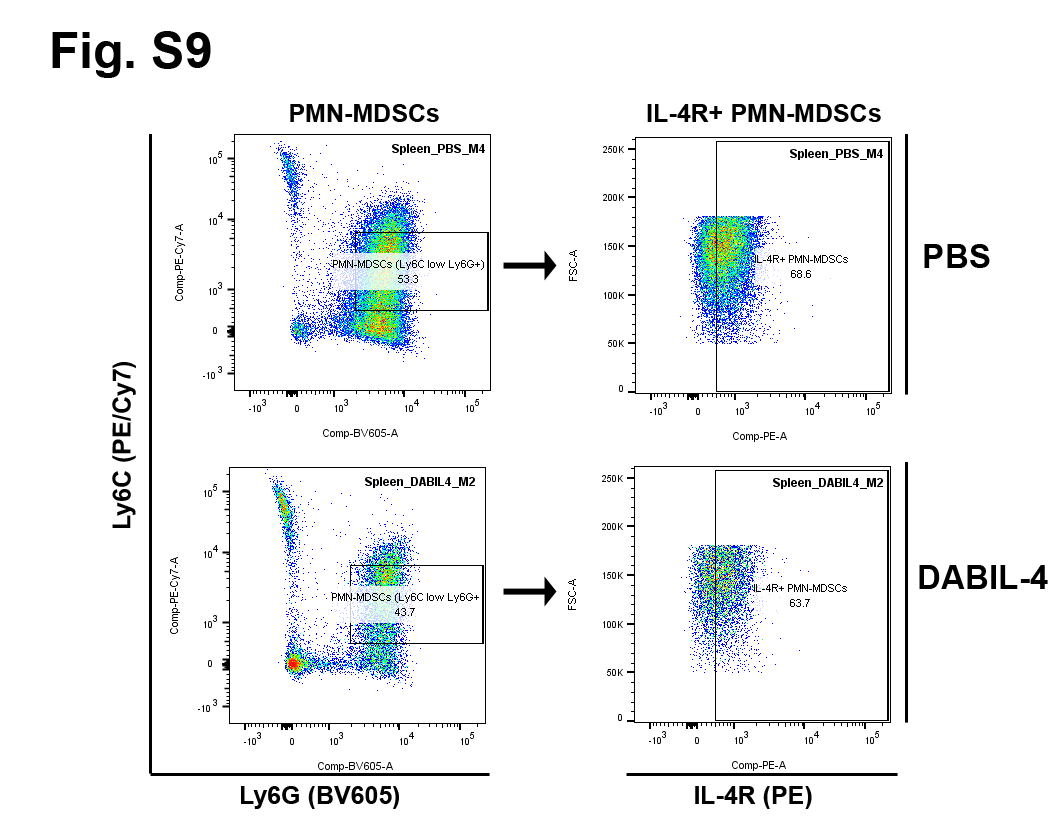

Supplement: Supplementary file 9 — Fig. S9. Two‐dimensional representation of FACS data to demonstrate depletion of IL‐4R+ PMN‐MDSCs in spleen isolated from DABIL‐4 treated mice on day 25. [file MOL2-15-1330-s013.tif]

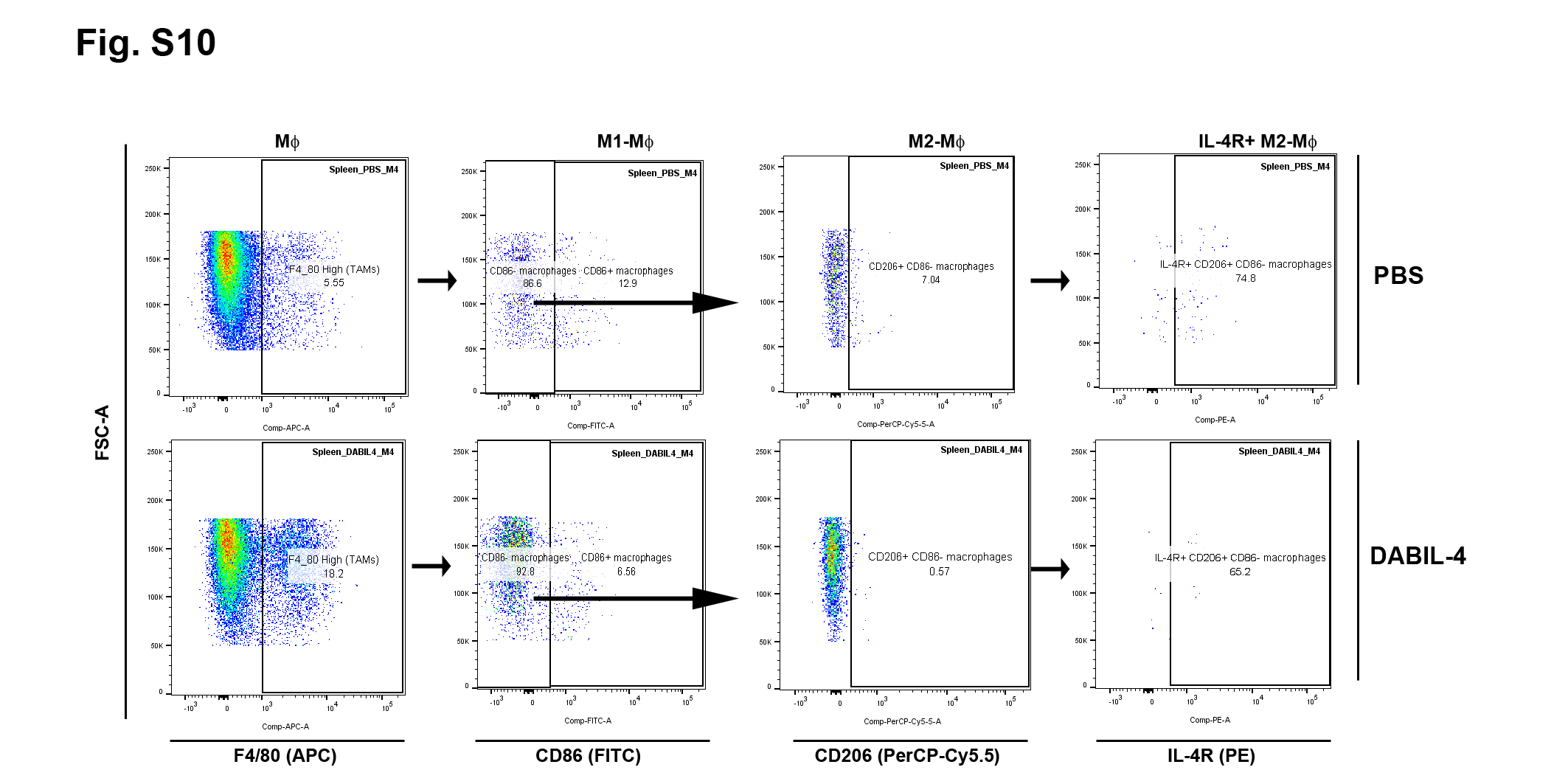

Supplement: Supplementary file 10 — Fig. S10. Two‐dimensional representation of FACS data to show depletion of M2 macrophages in spleens isolated from DABIL‐4 treated mice on day 25. [file MOL2-15-1330-s009.tif]

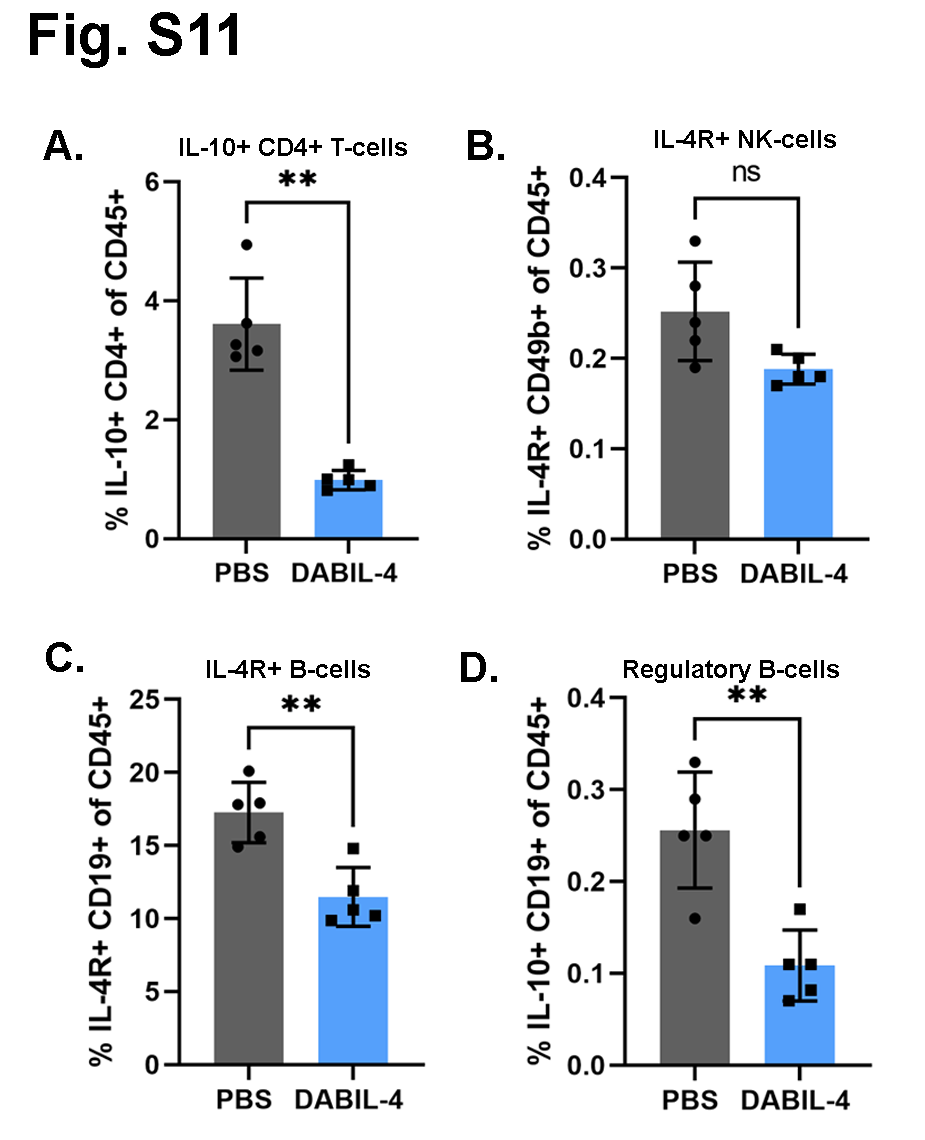

Supplement: Supplementary file 11 — Fig. S11. DABIL‐4 administration depletes IL‐4R+ B‐cells, Bregs and IL‐10+ CD4+ T‐cell populations in spleen. [file MOL2-15-1330-s003.tif]

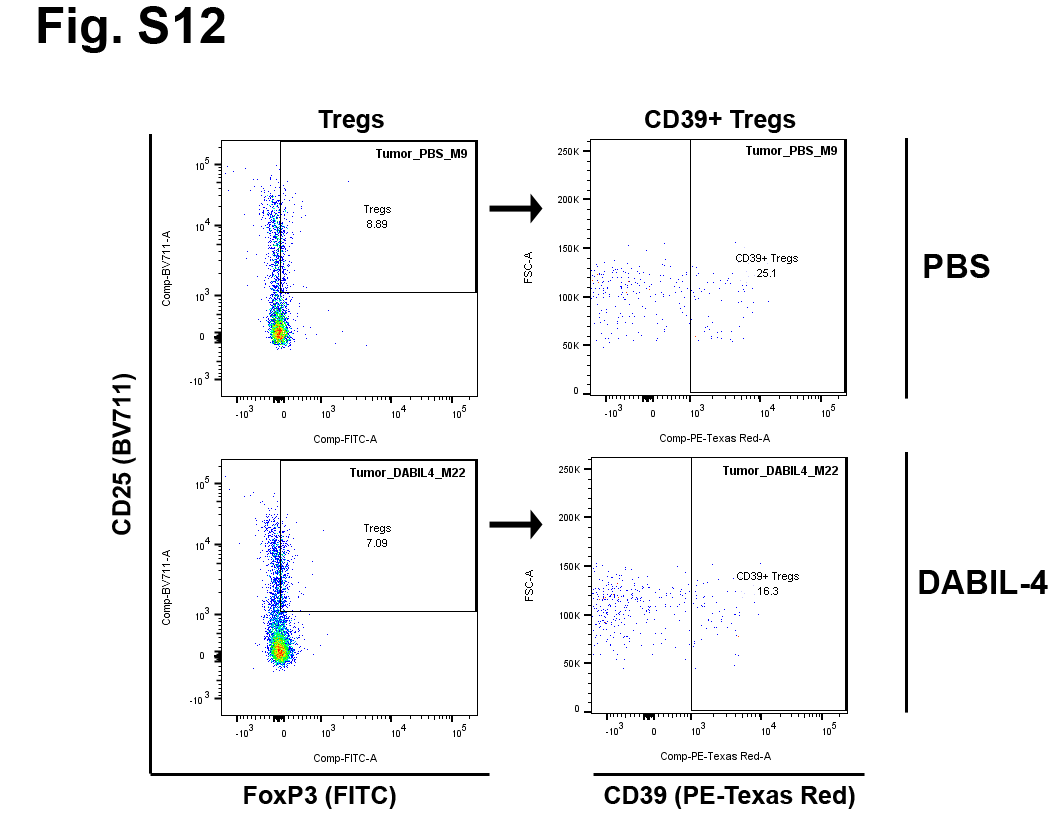

Supplement: Supplementary file 12 — Fig. S12. Two‐dimensional representation of FACS data to show depletion of CD39+ Tregs in tumors isolated from DABIL‐4 treated mice on day 17. [file MOL2-15-1330-s006.tif]
