# Supplementary material for: Therapeutic targeting with DABIL‐4 depletes myeloid suppressor cells in 4T1 triple‐negative breast cancer model
Source: Mol Oncol. 2021 Mar 24;15(5):1330–44. doi: 10.1002/1878-0261.12938 (PMC8096791; doi:10.1002/1878-0261.12938)
Supplement: Supplementary file 13 — Supplementary Material [file MOL2-15-1330-s007.docx]

**SUPPLEMENTARY INFORMATION**

**Therapeutic targeting with DABIL-4 depletes myeloid suppressor cells in 4T1 triple-negative breast cancer model**

Sadiya Parveen^1^, Sumit Siddharth^2^, Laurene S Cheung^1^, Alok Kumar^1^, Jessica Shen^1^, John R Murphy^1*^, Dipali Sharma^2*^, and William R Bishai^1*^

^1^Department of Medicine, Division of Infectious Diseases, Johns Hopkins University School of Medicine, Baltimore, MD 21231.

^2^Department of Oncology, Sidney Kimmel Comprehensive Cancer Center at Johns Hopkins, Johns Hopkins University School of Medicine and the, Baltimore, MD 21231, USA.

*Correspondence to be addressed to: [john.murphy@jhmi.edu](mailto:john.murphy@jhmi.edu), [dsharma7@jhmi.edu](mailto:dsharma7@jhmi.edu), [wbishai@jhmi.edu](mailto:wbishai@jhmi.edu)

**This PDF file includes:**

Figures legends for S1 to S12

**FIGURE LEGENDS**

**Fig S1.** pKN2.6Z-LC128 shuttle vector plasmid map.

**Fig S2. Genetic construction and purification of DABIL-4 fusion toxin using *C. diphtheriae*. (A)** Schematic of DABIL-4 fusion toxin construct compared to DABIL-2 **(B)** Coomassie-stained SDS/PAGE gel after purification (lane 2). Lane 1 is molecular weight markers. Expected size of DABIL-4 is 58 kDa. **(C)** Immunoblot of the purified DABIL-2 and DABIL-4 fusion toxins. Staining with anti-DT antibody revealed diffuse bands for both DABIL-2 and DABIL-4 and arrow corresponds to the molecular weight of the fusion toxins. The experiment was performed at least three times.

**Fig S3. Cytotoxic activity of DABIL-4 against NT2.5 cells tested using MTS-based assay.** Data is shown as mean ± SD. The experiment was performed in triplicates.

**Fig. S4. DABIL-4 induces apoptosis in IL-4R+ tumor cells. (A)** Caspase 3/7 activity luminescence-based assay performed with 4T1 cells treated with 20 nM DABIL-4. Caspase activity was comparable to doxorubicin (Dox), an agent known to cause apoptosis in 4T1 cells**.** Data is shown as mean ± SEM. **(B)** Immunoblot analysis of 4T1 cells treated with varying concentration of DABIL-4 showed upregulation of apoptotic markers (cleaved PARP and cleaved Caspase-3). β-actin was used as the loading control. The experiments were performed in triplicates. Statistical significance was calculated by two-tailed unpaired student t-test considering an unequal distribution. **P < 0.01.

**Fig S5. DABIL-4 administration induces apoptosis in 4T1 tumors in vivo**. **(A)** Immunohistochemical staining of 4T1 breast cancer tumor tissue (from 3 mice) for induction of apoptosis using cleaved caspase 3 antibody. The apoptotic tumor cells are stained brown. 4T1 tumors from both PBS- and DABIL-4 treated groups were harvested on day 25 post tumor-implant. The size of the scale bar is 100 µM. **(B)** Flow cytometry analysis of tumors (n=4 per group) on day 22 post tumor-implant, showed robust IL-4R expression upon tumor cells and DABIL-4 treatment reduced the frequency of IL4R^+^ tumor cells. For the identification of the tumor, we gated live cells to identify CD45^-^ CD3^-^ CD11b^-^ CD124^+^ cells. Data is represented as mean ± SD. Statistical significance was calculated by two-tailed unpaired student t-test considering an unequal distribution. Data are shown as mean ± SD. **P < 0.01.

**Fig S6. DABIL-4 exhibits anti-tumor activity in E0771 adenocarcinoma model in C57BL/6 mice.** 50,000 E0771 cells were orthotopically implanted in C57BL/6 mice (n=5 per group). Starting day 11, DABIL-4 was given i.p. on alternate days for total of 5 doses. Tumor volumes were measured using electronic Vernier Calipers. Statistical significance was calculated by two-tailed unpaired student t-test considering an unequal distribution. Data are shown as mean ± SD. *P < 0.05, **P < 0.01.

**Fig S7. Quantification of lung metastases using IVIS bioluminescence imaging.** 10,000 4T1-luc cells were orthotopically implanted in mammary fat pad of syngeneic Balb/c mice (n=3 per group). After 12 days, primary tumors were surgically removed and mice were treated with 10 µg DABIL-4 every third day. On day 9 post tumor removal, thoracic cavity of the mice was imaged using IVIS-imaging (Perkin-Elmer) and total bioluminescence was calculated. **(A)** The images of the thoracic cavity of both PBS- and DABIL-4 treated mice and, **(B)** total photon flux resulting from the thoracic cavity bioluminescence is shown here. Data is plotted as mean ± SEM. Statistical significance between the groups was assessed by two-tailed unpaired student t-test considering an unequal distribution. No statistical significance was observed.

**Fig S8. DABIL-4 treatment modulates myeloid cell populations in spleen and tumor microenvironment.** Single cell suspensions of spleen and tumors were stained and analyzed by flow cytometry (n=5). We evaluated differences in the population of **(A)** IL-4R^+^ MDSCs of CD45^+^, **(B)** IL-4R^+^ M-MDSCs of CD45^+^, (**C)** TNFα^+^ macrophages of CD45^+^and, **(D)** IL-4R^+^ CD206^+^ expression on macrophages. All panels correspond to day 25 post tumor implantation. Statistical significance between the groups was assessed by two-tailed unpaired student t-test considering an unequal distribution. Data are represented as mean ± SD. *P < 0.05, NS=non-significant.

**Fig S9. Two-dimensional representation of FACS data to demonstrate depletion of IL-4R+ PMN-MDSCs in spleen isolated from DABIL-4 treated mice on day 25.** Doublets and debris were excluded. Live CD45+ CD11b+ cells were gated using Ly6G and Ly6C to identify PMN-MDSCs subset; which was gated further to identify IL-4R+ PMN-MDSCs subsets.

**Fig S10. Two-dimensional representation of FACS data to show depletion of M2 macrophages in spleens isolated from DABIL-4 treated mice on day 25.** Doublets and debris were excluded. Live CD45+ CD11b+ cells were gated to identify F4/80+ macrophages; which were then gated into CD86+ M1 macrophages. CD86- macrophages were further gated to identify CD86- CD206+ M2 macrophages; which was further gated to identify IL-4R+ M2 macrophage subset.

**Fig S11. DABIL-4 administration depletes IL-4R+ B-cells, Bregs and IL-10+ CD4+ T-cell populations in spleen.** As in **Fig 2A**, mice were treated with DABIL-4 thrice weekly beginning on day 7, and they were sacrificed on day 15. Single cell suspensions of spleens from both treatment groups were stained with specific antibodies and analyzed by flow cytometry (n=5). We analyzed differences in the population of **(A)** IL-10^+^ CD4^+^ T-cells, **(B)** IL-4R^+^ NK-cells, **(C)** IL-4R^+^ B-cells and, **(D)** IL-10^+^ B-cells also known as regulatory B-cells (Bregs). Data are represented as mean ± SD and as percentage of CD45^+^ population. Spleen were analyzed on day 15 post tumor implant. Statistical significance between the groups was assessed by two-tailed unpaired student t-test considering an unequal distribution. *P < 0.05, **P < 0.01, ***P < 0.001, ****P < 0.0001.

**Fig S12. Two-dimensional representation of FACS data to show depletion of CD39^+^ Tregs in tumors isolated from DABIL-4 treated mice on day 17.** Doublets and debris were excluded. Live CD45+ CD3+ CD4+ CD8- cells gated using CD25 and FoxP3 markers to identify Tregs; Tregs were further gated using CD39 marker to identify activated Tregs population.
